# Role of mucin glycosylation in the gut microbiota-brain axis of core 3 *O*-glycan deficient mice

**DOI:** 10.1038/s41598-023-40497-8

**Published:** 2023-08-26

**Authors:** Erika Coletto, George M. Savva, Dimitrios Latousakis, Matthew Pontifex, Emmanuelle H. Crost, Laura Vaux, Andrea Telatin, Kirk Bergstrom, David Vauzour, Nathalie Juge

**Affiliations:** 1 Gut Microbes and Health Institute Strategic Programme, Quadram Institute Bioscience, Norwich, NR4 7UQ UK; 2 Norwich Medical School, Biomedical Research Centre, University of East Anglia, Norwich Research Park, Norwich, NR4 7TJ UK; 3 Department of Biology, University of British Columbia, Okanagan Campus, 3333 University Way, Kelowna, BC V1V 1V7 Canada

**Keywords:** Microbiology, Neurology

## Abstract

Alterations in intestinal mucin glycosylation have been associated with increased intestinal permeability and sensitivity to inflammation and infection. Here, we used mice lacking core 3-derived *O*-glycans (C3GnT^−/−^) to investigate the effect of impaired mucin glycosylation in the gut-brain axis. C3GnT^−/−^ mice showed altered microbial metabolites in the caecum associated with brain function such as dimethylglycine and *N*-acetyl-l-tyrosine profiles as compared to C3GnT^+/+^ littermates. In the brain, polysialylated-neural cell adhesion molecule (PSA-NCAM)-positive granule cells showed an aberrant phenotype in the dentate gyrus of C3GnT^−/−^ mice. This was accompanied by a trend towards decreased expression levels of PSA as well as ZO-1 and occludin as compared to C3GnT^+/+^. Behavioural studies showed a decrease in the recognition memory of C3GnT^−/−^ mice as compared to C3GnT^+/+^ mice. Combined, these results support the role of mucin *O*-glycosylation in the gut in potentially influencing brain function which may be facilitated by the passage of microbial metabolites through an impaired gut barrier.

## Introduction

The gastrointestinal (GI) tract hosts a complex microbial community of bacteria, archaea and eukarya, collectively known as the gut microbiota which influences the balance between health and the onset of intestinal and extra intestinal diseases^1^. In recent years, it has become increasingly clear that the gut microbiome is involved in gut-brain signalling, leading to the emergence of a gut microbiota-brain axis concept^2,3^. Mounting preclinical evidence broadly suggests that the gut microbiota can modulate brain development, function, and behaviour through immune, metabolic, and neuronal pathways^4^. During dysbiosis, these pathways are dysregulated and associated with altered permeability of the blood–brain barrier (BBB) and neuroinflammation^5^.

The structure and function of the gut microbiota varies along the GI tract, but also from the lumen to the mucosa^6^. In the colon, mucus covering the epithelium is critical to gut homeostasis by harbouring a microbial community at safe distance from the epithelium surface^7^. Intestinal mucin *O*-glycans, the main structural components of mucus, provide binding sites and a sustainable source of nutrients to the bacteria inhabiting the mucus niche^8–10^, therefore contributing to the spatial organisation of the gut microbiota^11,12^. Mucin *O*-glycosylation is initiated by the addition of a *N*-acetylgalactosamine (GalNAc) residue to the hydroxyl group of serine or threonine, resulting in the formation of the Tn antigen, which represents the substrate for further additions of sugars by glycosyltransferases. The addition of galactose (Gal) to the Tn antigen results in the formation of core 1 (Galβ1-3GalNAcα-Ser/Thr) whereas addition of *N*-acetylglucosamine (GlcNAc) to the Tn antigen results in the core 3 (GlcNAc-β1-3GalNAcα-Ser/Thr) structure. The further extension of core 1 and core 3 with GlcNAc residues by the action of β1,6 *N*-acetylglucosaminyltransferase gives rise to the core 2 (Galβ1,3(GlcNAcβ1,6)GalNAcα1-Ser/Thr) and core 4 (GlcNAcβ1,6(GlcNAcβ1,3)GalNAcαSer/Thr) structures, respectively^13^. The distribution of the mucin core structures varies along the GI tract, which is partly due to the organ-specific expression patterns of the core glycosyltransferases^14^. Core 1 and 2 structures are typical of gastric and duodenal mucins, whereas core 3 and core 4 are abundant in the colonic mucins^14–16^. Under homeostatic conditions, these mucin core structures are further elongated by the addition of monosaccharides^17^ and the mucin glycan chains are usually terminated with sulfate, sialic acid or fucose residues, resulting in highly diverse oligosaccharide structures^13,18,19^.

Modification in mucin *O*-glycosylation causes a disruption of host–microbe interactions and mucosal immunity, contributing to a compromised intestinal barrier and associated diseases such as inflammatory bowel diseases (IBD)^11,20–22^. Understanding the role of mucin glycosylation in health and diseases is challenging, due to the large diversity of *O*-glycan structures and enzymatic pathways regulating the *O*-glycosylation machinery^23^. However, in recent years, glycosyltransferase knockout mice, displaying defects in glycosylation, have been instrumental in demonstrating the causal role of *O*-glycans in several physiological processes. Mice lacking core 3-derived* O*-glycans (C3GnT^−/−^) showed increased susceptibility to chemically-induced colitis and colon cancer but unlike mice lacking intestinal core 1-derived *O*-glycans (IEC C1galt1^−/−^), they do not develop spontaneous colitis^20,24–26^. The defect of glycosylation in these mouse models led to a reduction of Muc2 protein and a thinner outer mucus layer which caused an increased intestinal permeability and higher responsiveness to infections, due to direct contact of the gut microbiota with the epithelium layer^24^. In addition, while C1galt1 is ubiquitously expressed, C3GnT is most highly expressed in the proximal colon^25^. Interestingly, the number of goblet cells remain unaltered in these transgenic animal models, suggesting that deficiency in mucin glycosylation is enough to disturb the intestinal epithelium barrier^26,27^.

A “leaky gut” may contribute to the key signalling pathways mediating the gut microbiota-brain axis^28^. A compromised intestinal barrier allows broader engagement of the immune system, but it also weakens the containment of microbial products, which can leak from the intestine into the circulation, resulting in chronic low-grade inflammation^2^ observed in neurological disorders^29^. Gut barrier function is preserved by multiple protective layers including the gut microbiota, the mucus layer, and epithelial and immune cells but the role of mucin glycosylation in gut microbiota-brain axis has not been investigated. Here, we used C3GnT^−/−^ mice as a model to investigate the effect of altered mucin glycosylation in the gut on brain function and neurological behaviour.

## Results

### C3GnT^−/−^ mice showed alterations in mucin glycosylation, gut barrier function and gut microbiota profile

**Figure 1:**
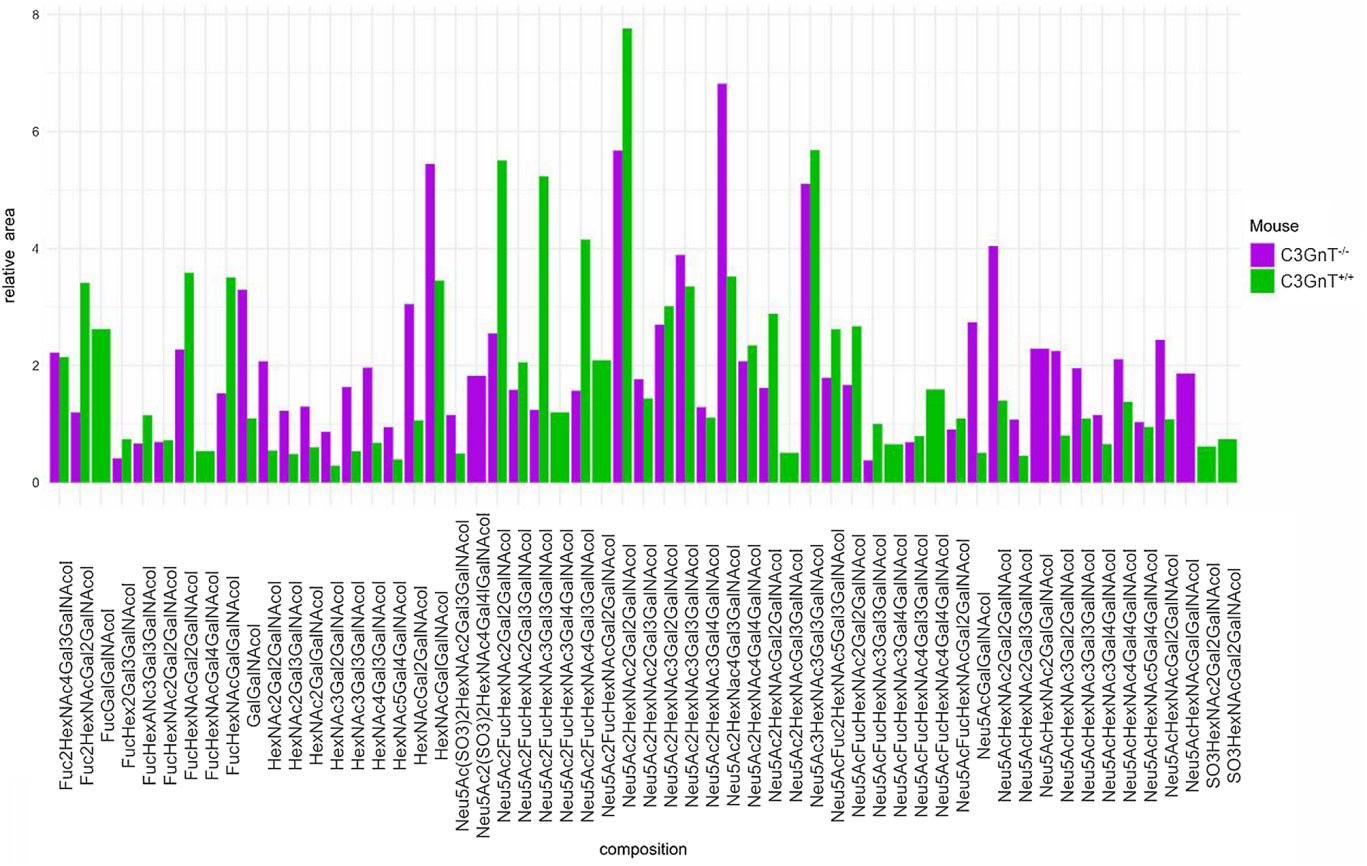
Relative abundance of glycans in colonic soluble mucins. Graph showing abundance of glycans of different composition in soluble mucins from C3GnT^−/−^ and C3GnT^+/+^ mice.

Previous work showed that deletion of the C3GnT gene (coding for core 3 beta1,3 *N*-acetylglucoaminyltransferase also known as B3GNT6 UDP-GlcNAc:betaGal beta-1,3-*N*-acetylglucosaminyltransferase 6) eliminated core 3-derived *O*-glycans and significantly reduced total intestinal *O*-glycans^26^ with Muc2 from C3GnT^−/−^ mice carrying a mixture of core 1- or core 2-type glycans^13^. Here, we first confirmed that the C3GnT gene was mainly expressed in the proximal colon of the C3GnT^+/+^ mice, as compared to C3GnT^−/−^ mice, with no expression detected in the brain of both genotypes (Fig. S1). We then investigated the relationship between changes in mucin glycosylation and gut microbiota profile in C3GnT^−/−^ and C3GnT^+/+^ littermates. The glycosylation profile of purified mucins from littermates was analysed by MALDI-TOF following release of glycans by reductive β-elimination, and permethylation. The analysis confirmed that the lack of core 3 *O*-derived glycans affected the relative abundance of *O*-glycan composition in colonic soluble mucins (Fig. 1). The abundance of glycans was then determined as the ratio of the area of each identified glycan peak over the sum of all identified glycan peaks per sample. Overall, the percentage of sialylated glycans was similar between C3GnT^−/−^ mice and C3GnT^+/+^ mice (71.1% for C3GnT^−/−^ vs 69.2% for C3GnT^+/+^), whereas a marked decrease was observed in the abundance of fucosylated glycans in the C3GnT^−/−^ mice (21.4%) as compared to C3GnT^+/+^ mice (49.7%). There was low sulfation percentage in both groups of mice (1.85% for C3GnT^+/+^ and 2.98% for C3GnT^−/−^). The fragmentation spectra of glycans also showed altered levels of glycan structures, other than core 3 or core 4, as the lack of core 3 *O*-derived glycans prevented the further synthesis of core 4 glycans^30^. For example, the glycan peak at 1590 Da corresponding to glycans composed of one Neu5Ac, three *N*-acetyl-hexosamines and two hexose units was found in both C3GnT^+/+^ and C3GnT^−/−^ but there were marked differences in the fragmentation spectra, such as the presence of the m/z 694 peak in the C3GnT^+/+^ sample and the m/z 955 and 1314 peak in the C3GnT^−/−^ sample, indicating differences in linkages between monosaccharides of these glycan structures (Fig. S2).

**Figure 2:**
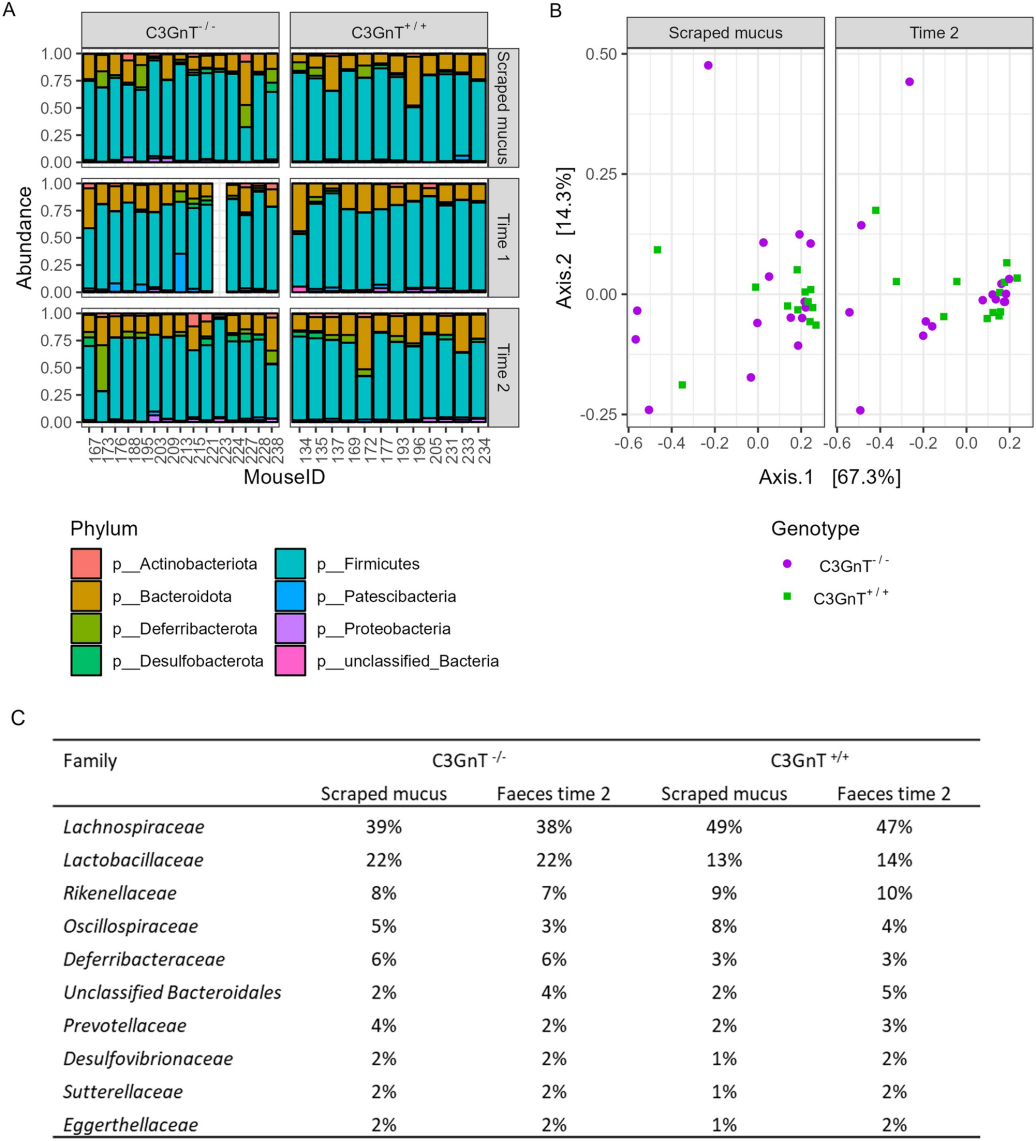
Gut microbiota composition from scraped mucus and faecal samples of C3GnT^−/−^ and C3GnT^+/+^ mice. (**A**) Bar chart of gut microbiota composition at the phylum level from scraped mucus at Time 2, and faecal pellet at Time 1 and Time 2 of C3GnT^−/−^ (n = 12) and C3GnT^+/+^ (n = 15) mice. (**B**) PCoA ordination plot with Bray–Curtis dissimilarity (C) Table indicating the most abundant bacterial families grouped by genotype (C3GnT^−/−^ and C3GnT^+/+^) and expressed by percentages in the scraped mucus and faecal pellet at Time 2.

The gut microbiota from colonic scraped mucus (also used for the mucin glycosylation profile) and faecal samples of C3GnT^−/−^ mice and C3GnT^+/+^ mouse littermates were analysed by sequencing the 16S rDNA variable region V3-V4. At the phylum-level, the dominant components were members of Firmicutes/Bacillota and Bacteroidetes/Bacteroidota with no major differences in phyla between C3GnT^−/−^ and C3GnT^+/+^ mice, either in faecal samples at 21 (Time 1) and 75 days (Time 2) after birth or in scraped mucus (Fig. 2A). There was no evidence for any difference in α-diversity between groups in faecal samples or in mucus (observed OTU counts, Shannon index and Simpson’s index). The β-diversity of the faecal samples and mucus samples was calculated using both Unifrac and Bray–Curtis distance, and ordinations were plotted, stratified by sample point for each measurement. PERMANOVA showed no evidence for differences in composition between groups at any sample point (Fig. 2B). When comparing abundances at family level, *Lachnospiraceae* and *Lactobacillacea* were the most dominant families in the mucus and in the 75 days faecal pellet of both C3GnT^−/−^ and C3GnT^+/+^ mice (Fig. 2C). Averaging across sample points, 48% of reads were attributed to *Lachnospiraceae* in C3GnT^+/+^ mice, as compared to 38% in C3GnT^−/−^ mice. There was a corresponding increase in *Lactobacillaceae* in C3GnT^−/−^ mice where the relative abundance was 22% versus 14% in C3GnT^+/+^ mice (Fig. 2C, Table S1). Analysis using Deseq2 provided some evidence that the relative abundance of *Lactobacillaceae* in 75-day faecal samples was higher in the C3GnT^−/−^ after multiple testing correction (log2 fold-change = 1.7; p = 0.004; q = 0.085), with a similar magnitude of difference seen in the mucus samples (log2 fold-change = 1.56; p = 0.027; q = 0.31). However, these differences were not statistically significant when compared using alternative approaches for differential abundance including linear regression analysis of relative abundances or centred log-ratios.

In line with previous results, we showed an increase in intestinal permeability in C3GnT^−/−^ compared to C3GnT^+/+^ littermates using FITC-dextran administered by oral gavage, supporting the role of core-3 derived *O*-glycans in the maintenance of mucosal integrity^26^ (Fig. S3).

### C3GnT^−/−^ mice showed alteration in granule cells morphology and PSA-NCAM expression in the brain

**Figure 3:**
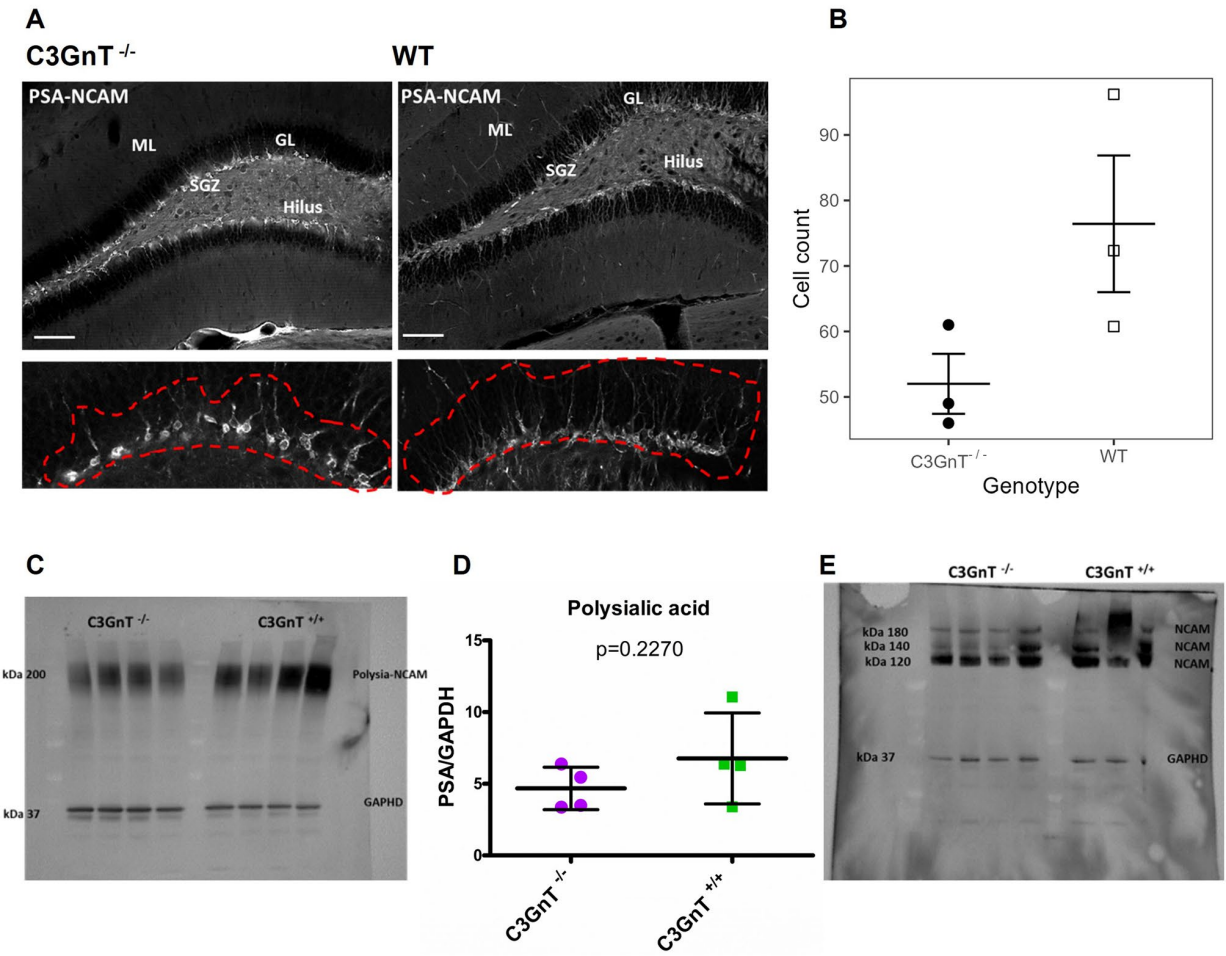
Analysis of PSA-NCAM in the brain of C3GnT^+/+^ and C3GnT^−/−^ littermates. (**A**) Immunohistochemistry images showing the phenotype of PSA-NCAM^+^ cells in the DG of C3GnT^−/−^ and WT mice. A detailed fraction of the dorsal part of the DG (red dashed line) shows the phenotype of granule cells in C3GnT^−/−^ mice exhibiting shorter and disorganised dendrites departing from an enlarged cell soma (bottom left), as compared to WT mice where the typical polarised morphology with fully developed arborisation departing from soma cells is shown. (**B**) PSA-NCAM^+^ cells appear decreased in the DG of C3GnT^−/−^ although this difference is not statistically significant (p = 0.13). Results are presented as mean ± SEM. PSA-NCAM^+^ cells were counted in 4 to 7 slides per animal. Images: objective 10×, scale bar: 100 µm. For the Western Blot analysis, the lysates were separated by 4–12% SDS-PAGE with 50 µg of total protein per lane. Western blot membrane showing (**C**) PSA expression in brain extract and (**D**) quantification carried out using ImageLab software. (**E**) Western blot membrane showing NCAM isoform pattern following endosialidase treatment. GAPDH was used as control.

To determine the consequence of altered intestinal mucin *O*-glycosylation barrier function on the neural plasticity occurring in the hippocampus, we focused on the phenotype of granule cells expressing PSA-NCAM in the dentate gyrus (DG) of C57BL/6 wild-type (WT) and C3GnT^−/−^ mice. Between 4 and 7 sections per mouse (from − 1.58 mm rostral/dorsal to − 2.54 mm caudal/ventral Bregma position) were analysed in each of the two groups (Fig. S4). From the dorsal to the ventral position of the hippocampus, the PSA-NCAM-positive (PSA-NCAM^+^) immature granule cells, proliferating cells supporting the neurogenesis in the DG^31,32^, showed an aberrant phenotype in the C3GnT^−/−^ mice, with short and disorganised neurites projecting towards the upper molecular layer whereas, in WT mice, the PSA-NCAM^+^ cells showed a typical polarised morphology with extensive apical dendrite branches reaching the above molecular layer (Fig. 3A). When performing a comparative counting of the PSA-NCAM^+^ granule cells in the subgranular zone (SGZ), the C3GnT^−/−^ mice had fewer positive cells per section (mean average = 52.0, s.e. = 8.0) compared to WT mice (mean average = 76.4, s.e. = 8.0) although not statistically significant (p = 0.09) when further analysed using a linear mixed model with random effect of mouse (Fig. 3B). However, this observed dysregulation of PSA-NCAM^+^ granule cells in the DG of C3GnT^−/−^ mice was in line with the decrease in polysialic acid content in the brain lysates of C3GnT^−/−^ mice as compared to C3GnT^+/+^ littermates as determined by immunoblot (Fig. 3C) and quantified by analysis of band intensity (with mean value intensity of 11 for C3GnT^−/−^ and 21 for C3GnT^+/+^) (Fig. 3D), whereas no alteration of NCAM expression levels or isoform pattern was observed (Fig. 3E).

**Figure 4:**
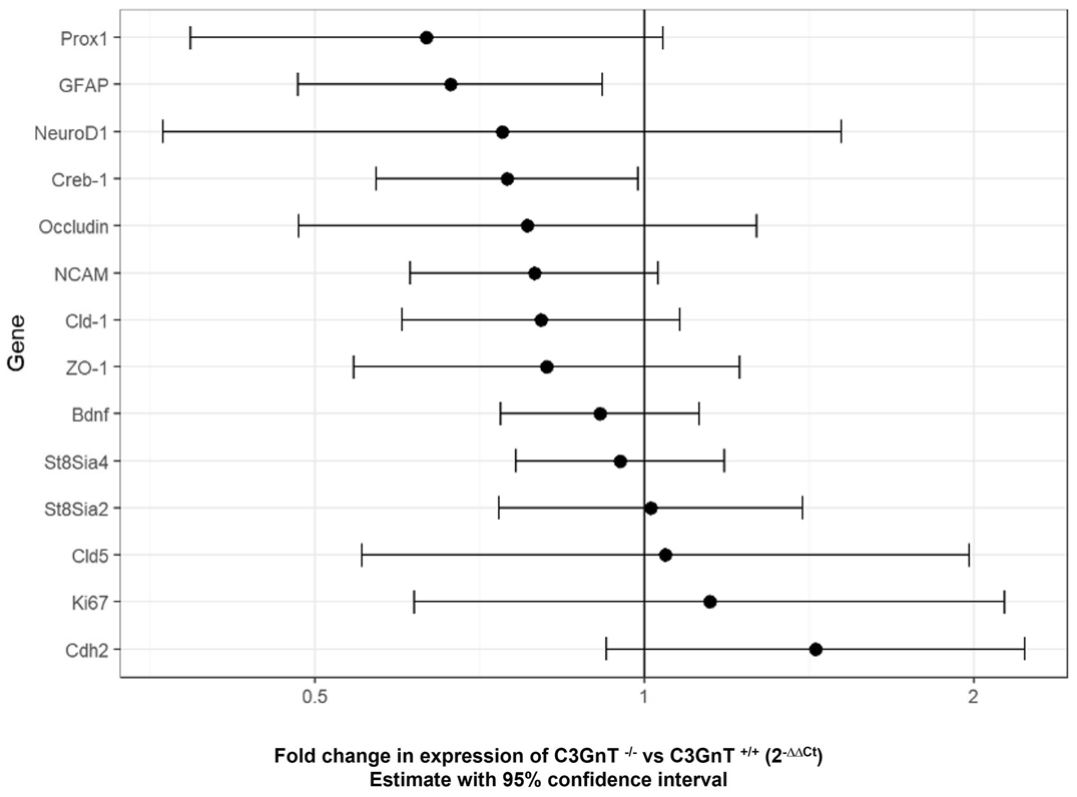
Gene expression analysis of gene targets in the brain of C3GnT^+/+^ and C3GnT^−/−^ littermates. Analyses were performed by RT-PCR. Effect size is the 2 to the power of the difference in Ct between genotypes, adjusted for Ct of housekeeping genes *Gapdh* and *Tbp*. This is interpreted as the fold change in expression between genotypes, with low values suggesting a higher expression in the C3GnT^+/+^ and high values corresponding to higher expression in C3GnT^−/−^. Error bars represent 95% confidence intervals.

**Figure 5:**
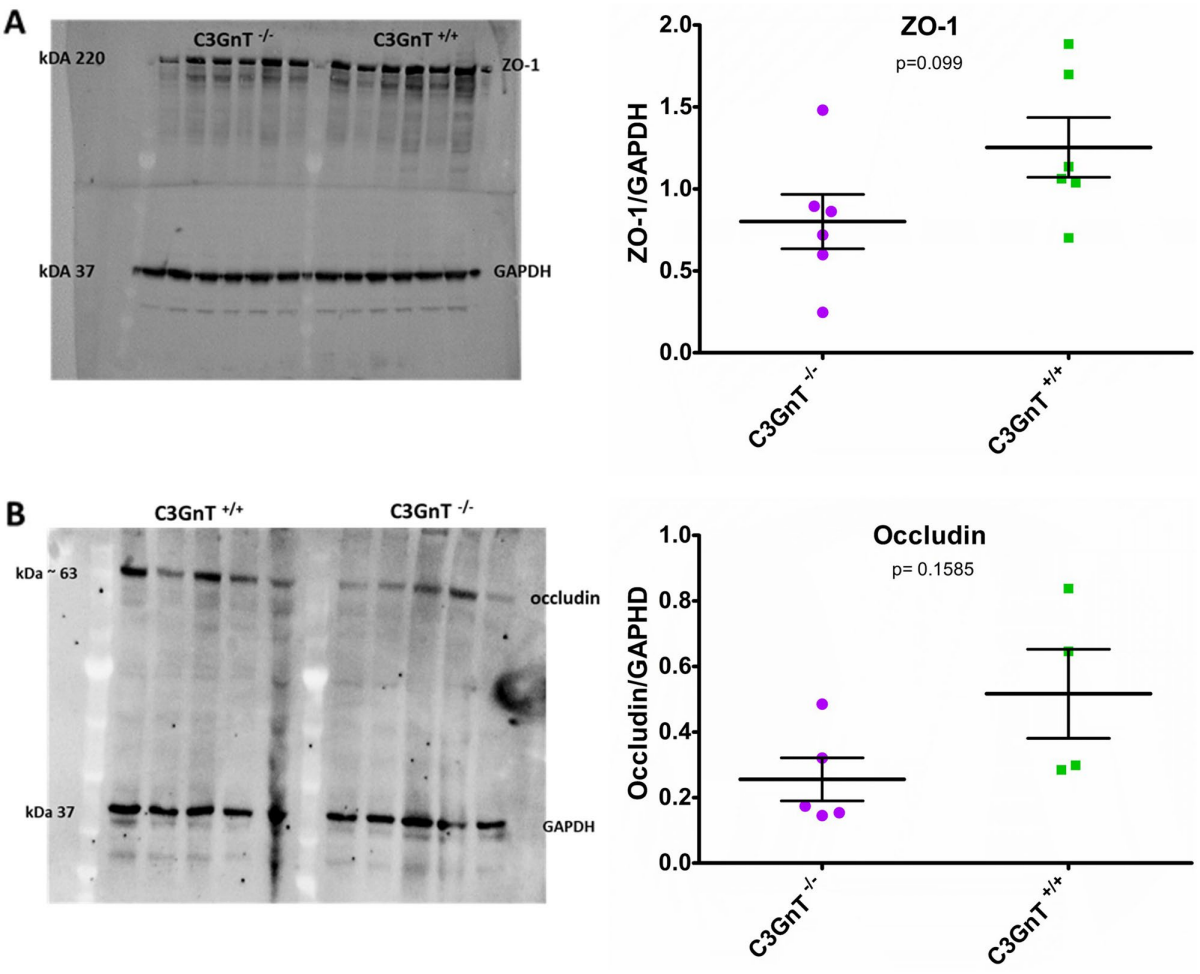
Analysis of tight junction proteins in the brain of C3GnT^+/+^ and C3GnT^−/−^ littermates. Proteins from brain lysates were separated using a 4–12% SDS-PAGE with 50 µg of total protein per lane. Western blot analysis showed (**A**) ZO-1 and (**B**) occludin. Quantification was carried out using ImageLab software. GAPDH was used as a control.

Having established that the morphology of PSA-NCAM^+^ granule cells in the DG of C3GnT^−/−^ mice appeared to be disorganised and atypical as compared to WT mice, we next investigated how this may relate to developmental process, synaptic plasticity and barrier permeability in the brain of C3GnT^−/−^ and C3GnT^+/+^ littermates. Gene expression analysis was carried by RT-qPCR using validated primers against a panel of 14 target genes and two housekeeping genes (*Gapdh*, *Tbp*). The gene expression differences were adjusted for both the housekeeping genes. The genes of interest were chosen as an indication of synaptic plasticity, neurodevelopment and proliferation (*BDNF*, *Creb1*, *NCAM1*, *Ki67*), polysialyltranferase activity (*ST8sia2*, *ST8sia4*), formation of tight junction and cadherin (*ZO-1*, *Cldn1*, *Ocln*, *Cdh2*), granule cell maturation (*GFAP*, *Prox1*, *NeuroD1*) (Fig. S5). Fold change of expression for each gene in C3GnT^−/−^ and C3GnT^+/+^ littermates are reported with 95% confidence intervals (Fig. 4). There was evidence for C3GnT^−/−^ showing a downregulation of cAMP Responsive Element Binding Protein 1 (*Creb1*, FC = 0.748, p value = 0.04) and of genes associated with the proliferative status of granule cells, such as glial fibrillary acidic protein (*Gfap*, FC = 0.664, p value = 0.015) although, significance was lost after correcting p-value for multiplicity using Benjamini and Hochberg method (Fig. 4). Western blot analysis showed a trend towards reduction in the expression of tight junction proteins ZO-1 (Fig. 5A) and occludin (Fig. 5B) in the brain of C3GnT^−/−^ as compared to the C3GnT^+/+^ mice, although quantification was not statistically significant at the protein level.

### C3GnT^−/−^ mice displayed distinct metabolite signatures in the caecum

**Figure 6:**
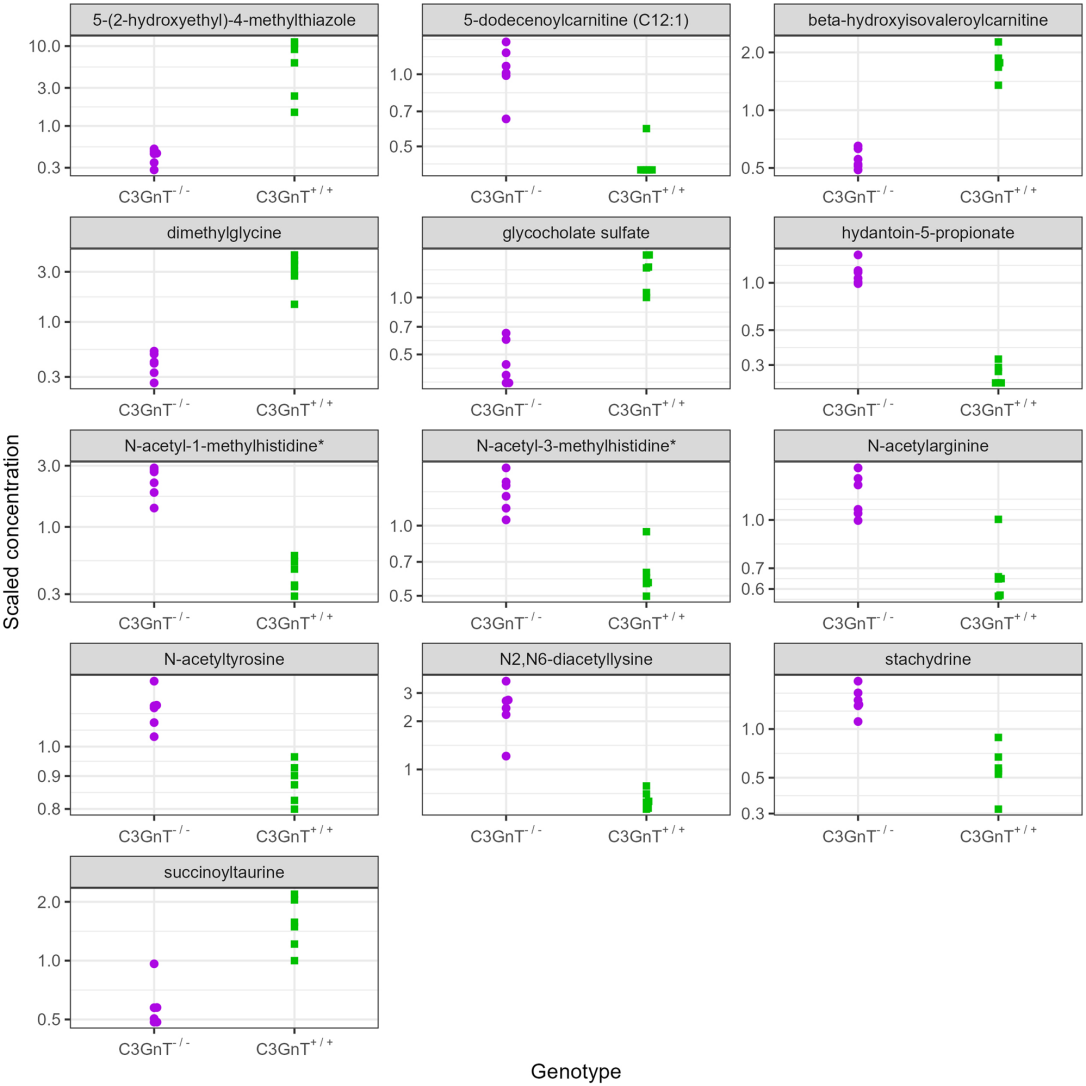
Metabolites in the caecum of C3GnT^+/+^ and C3GnT^−/−^ littermates. The plots show the distribution of log-concentrations of individual metabolites that were significant at q < 0.05 in the caecal content of the two mouse groups (log-concentrations are standardised to mean zero).

Untargeted metabolomics was carried out in order to investigate the range of metabolites affected by the altered mucin glycosylation. Principal Component Analysis (PCA), performed to obtain an overall comparative view of the metabolomics data from the brain and caecal content, showed some separation of the C3GnT^+/+^ and C3GnT^−/−^ samples in the brain (Fig. S6A) and a more distinctive segregation of C3GnT^−/−^ samples away from C3GnT^+/+^ samples in the caecal content (Fig. S6B). Primary analyses of the data showed differences in metabolites in the caecal content of C3GnT^+/+^ and C3GnT^−/−^ mice such as *N*-acetyl-lysine derived metabolites, acetyl-carnitines, dimethylglycine and acetyl-l-tyrosine (Fig. 6). The p-values were then adjusted for multiple testing correction using the Benjamini–Hochberg False Discovery Rate (FDR) correction and the metabolites stratified according to significant q < 0.05 p-values. This analysis showed 13 metabolites differed in concentration between genotypes and the most significant were generated by four main super-pathways including amino acid (n = 9), cofactor and vitamins (n = 1), lipid (n = 2), and xenobiotics metabolism (n = 1) (Fig. S7).

### C3GnT^−/−^ mice displayed an altered memory but no motor impairment

C3GnT^−/−^ and C3GnT^+/+^ mice were subjected to three different behavioural tests: the novel object recognition (NOR) that tests recognition memory, the Y-maze to assess spatial memory and the open field (OF) that analyses psychomotor functions and anxiety.

**Figure 7:**
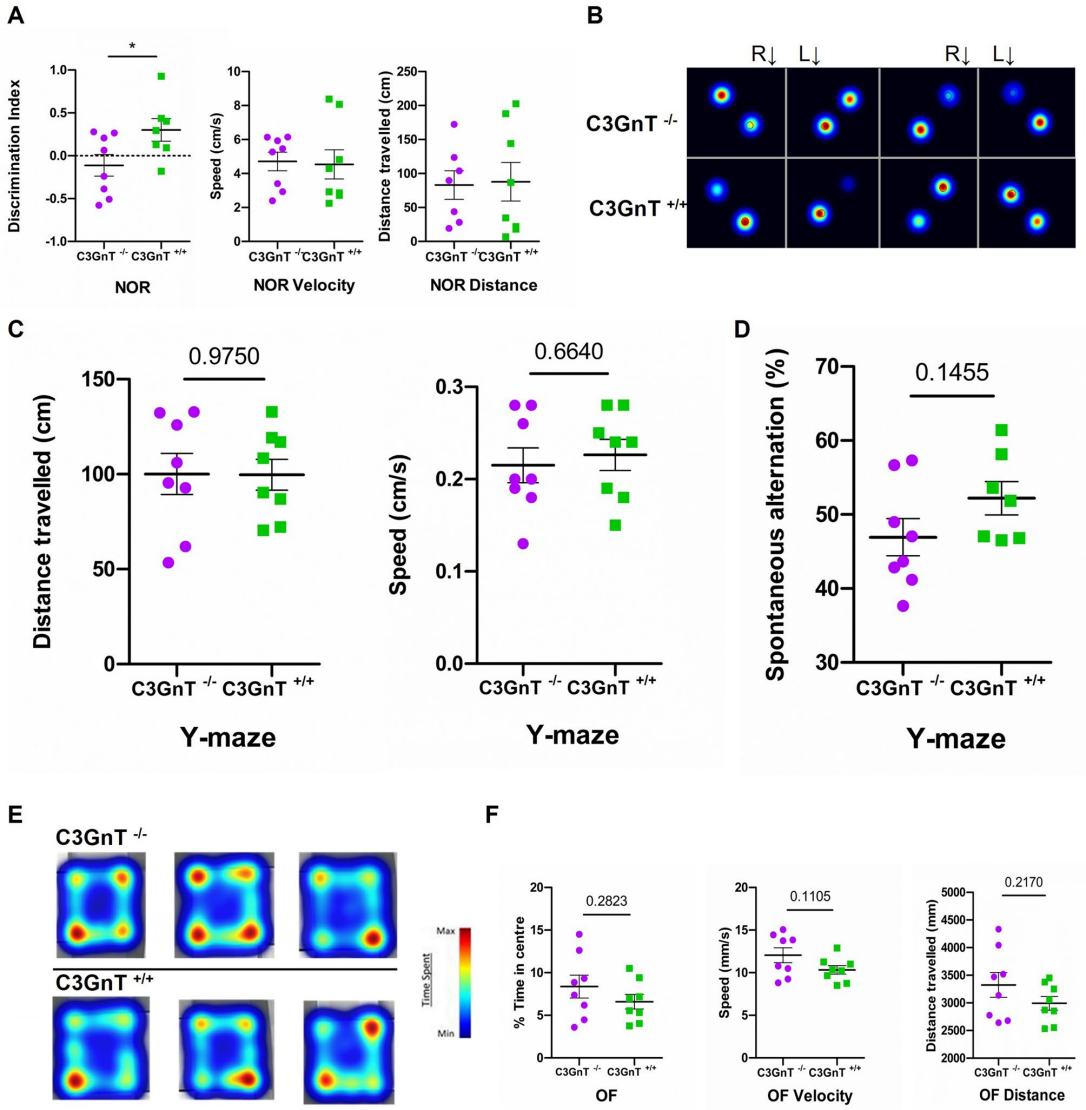
Behavioural tasks performed by C3GnT^+/+^ and C3GnT^−/−^ littermates. Novel object recognition (NOR). (**A**) The discrimination index, calculated as (TN − TF)/(TN + TF), was used to assess the preference for the novel object. Distance and speed of the task are also shown. Data are presented as mean ± SEM (n = 8/group) *p < 0.05. (**B**) The heat map visualisation tracks the time spent by the mice close to the novel object (black arrow) and the familiar object located in the opposite corner. Red represents increased time spent and blue represents minimal time spent during test. Y-maze (**C**) Scatterplots showing the distance travelled and speed in the three arms of the Y-maze by both mouse groups. (**D**) Scatterplot showing the spontaneous alternation in the Y-maze by both mouse groups. Data are presented as mean ± SEM (n = 8/group). Open field (OF) (**E**) The heat map visualisation showing typical examples of exploratory behaviour in the open-field test in the C3GnT^−/−^ and C3GnT^+/+^ mouse groups. Red represents increased time spent and blue represents minimal time spent during the test. (**F**) Scatterplots showing the total time spent in the centre of the box, the distance travelled in an open field and the speed during 5 min. Data are presented as mean ± SEM (n = 8/group).

The NOR test is an established behaviour test to evaluate memory alterations^33^, such as working memory, attention, anxiety and preference for novelty^34^. Following the habituation and familiarisation step with two identical objects, C3GnT^−/−^ and C3GnT^+/+^ mice were exposed to a novel object and a familiar object. If the memory of the testing subject is functioning normally, the mouse will spend more time exploring the novel object than exploring the familiar objects (Fig. S8A). If the exploration of all objects is the same, this behaviour will be indicative of a memory impairment^35^. The object recognition was assessed by a defined discrimination index calculated based on the time spent with the familiar and the novel object. Here, C3GnT^−/−^ mice showed a significant reduction in the time spent exploring the novel object (p = 0.0183, two-tailed unpaired t-test ± SEM) as compared to the C3GnT^+/+^ mice (Fig. 7A), suggesting an impairment of the recognition memory. As shown in the heatmap (Fig. 7B), C3GnT^−/−^ spent less time exploring the novel object in the maze, indicating a poor discrimination of the novel object as compared to the familiar one. The opposite was observed for the C3GnT^+/+^ mice, who spent more time with the novel object, exhibiting a typical exploratory behaviour.

The Y-maze can be used to assess motor-cognitive abilities and short-term memory in mice. The spontaneous alternation in the three arms measures the spatial working memory in the mice. A high percentage alternation is seen as a high proportion of entries into consecutive arms (Fig. S8B). A low percentage alternation (e.g. poor working memory) is seen as a higher proportion of repeated entries into the same arm. A mouse with a good working memory will remember the arms of the maze that it has already visited and will explore the less recently visited arm. This requires interaction across several different regions of the brain, such as the hippocampus and prefrontal cortex^36^. Here, no significant difference in the distance travelled by the C3GnT^−/−^ mice was observed when compared to C3GnT^+/+^ mice, indicating the lack of significant motor impairments of the animals (Fig. 7C). However, C3GnT^−/−^ mice showed a tendency to spend less time in the three arms of the maze compared to C3GnT^+/+^ mice, suggesting a slight worsening in spatial memory (Fig. 7D).

The open field is a common test to evaluate anxiety-like behaviour in animal models: it allows assessment of novel environment exploration, general locomotor activity, and provides an initial screen for anxiety-related behaviour in rodents^37^ (Fig. S8C). Rodents show distinct aversions to large, brightly open and unknown environments. In the open field test, when exposed to an open space, the mice generally exhibit thigmotaxis, which is the tendency to stay close to the walls of the open space and only later explore its centre^38,39^. Here, no differences between C3GnT^−/−^ and C3GnT^+/+^ mice were detected for the open field tasks, as shown in the heatmap where the distances travelled by mice of both genotypes appeared similar (Fig. 7E). However, C3GnT^−/−^ mice seemed to exhibit a more exploratory phenotype as indicated by increased speed and distance travelled in the box (Fig. 7F). Altogether the behavioural tests suggest a compromised hippocampal function as compared to C3GnT^+/+^ mice.

## Discussion

Glycans influence a variety of tissue development and pathophysiology aspects and alterations in glycosylation pathways contribute to the onset and/or progress of brain dysfunctions, from neurodevelopment disorders to neurodegenerative disorders^23,40^. Dysregulations of mucin-type *O*-glycosylation pathways in animal models have been shown to lead to a range of effects, from embryonic death to developmental defects and diseases such as colitis and cancer^41^. Core 3-derived *O*-glycans play an important role in the protective functions of mucus in the proximal colon where they are more highly expressed^25^. Here, we investigated the effect of an altered mucin glycosylation in the gut on brain physiology and behaviour.

We showed that C3GnT^−/−^ mice displayed decreased fucosylated glycans in colonic mucins consistent with the lack of core 3 glycans. Previous analyses showed an increase in total bacteria in mucus as compared to faecal samples in C3GnT^−/−^ mice and C1galt1^−/−^ mice (core 1 deficient mice)^26^. Here we showed that the phylogenic composition of the faecal and mucus-associated gut microbiota was stable after 3 weeks with Firmicutes and Bacteroidetes being the most abundant phyla, whereas Deferribacteres/Deferribacterota, Actinobacteria/Actinobacteriota/Actinomycetota and Proteobacteria/Pseudomonadota were less represented. These results are consistent with the dynamics of the gut microbiota in specific-pathogen free (SPF) mice, showing that at an early stage of life (9 days post weaning) and at late stage (141–150 days post weaning), the faecal microbiota is dominated by Firmicutes and Bacteroidetes with minor contribution from Actinobacteria, Proteobacteria and Verrucomicrobia/Verrucomicrobiota^42^*.* There were no clear differences in gut microbiota composition between the groups apart from an increase in the relative abundance of *Lactobacillaceae* in C3GnT^−/−^ mice which will need further confirmation. The increased intestinal permeability in C3GnT^−/−^ mice as compared to C3GnT^+/+^ mice may facilitate the passage of gut-microbe signals/metabolites to the brain, especially those derived from mucus-associated microbes which are in close proximity to the underlying mucosa. Here, we showed that the caecal content of C3GnT^−/−^ mice exhibited altered levels of metabolites linked with Alzheimer's disease (AD) and depression such as *N*-acetyl-lysine derived metabolites, acetyl-carnitines, dimethylglycine and acetyl-l-tyrosine^43–46^.

In the hippocampus, PSA-NCAM, expressed in two types of granule cells, intermediate progenitor cells and immature granule cells, was used as marker of proliferating neurons. Granule cells do not present PSA-NCAM when they become mature and enter the upper neural circuit^47,48^. Here, the PSA-NCAM^+^ immature granule cells in the neurogenic niche of the brain in the C3GnT^−/−^ mice showed an atypical phenotype with short and disorganised dendrites departing from the cell soma and an altered ratio between intermediate neural progenitor/immature granule cells, as compared to control mice where these cells exhibit a fully developed phenotype and a balanced distribution of the proliferating/differentiating granule cells^49^. This observation was supported by a reduced number of PSA-NCAM^+^ granule cells and a decrease in polysialic content in the brain of C3GnT^−/−^ mice as compared to the control mice. Given the role of PSA-NCAM^+^ granule cells in spatial and discrimination memory^50,51^, we next evaluated the behavioural pattern linked to memory and recognition in C3GnT littermates. This analysis revealed a significant alteration in the ability of C3GnT^−/−^ mice to discriminate novel and familiar objects as compared to C3GnT^+/+^ mice, suggesting an impairment of hippocampal memory circuits, consistent with the altered expression of PSA-NCAM in the DG of C3GnT^−/−^ mice, although involvement of the medial prefrontal cortex (mPFC) may also be considered, as previously reported^52^. The depletion of PSA-NCAM from the DG has been shown to reduce spatial learning and affect the long-term memory of non-spatial tasks in mice^53^. Furthermore, impairment of granule cells in the hippocampus are strongly linked with memory maintenance^54^ affecting the recognition memory task in rats^55^. In pre-weaning mice, a recent study reported that transgenic mice lacking the St6Gal1 gene encoding for β-galactoside α-2,6-sialyltransferase 1, the enzyme responsible of catalysing the transfer of sialic acid from CMP-sialic acid to galactose-containing substrates, showed an impaired spatial and recognition memory, a downregulation of the pathway involved in the formation of PSA-NCAM as well as an alteration in Firmicutes composition^56^, due to lack of 2,6-sialylation of milk oligosaccharides. Our data further support the importance of host glycosylation in mediating gut microbiota-brain communication.

In addition to the regulation of PSA-NCAM and the change in cognition in C3GnT^−/−^ mice, we observed a trend in decreased brain concentration of ZO-1 and occludin. These are two important proteins involved in the regulation of the blood–brain barrier^57^. For example, reduced expression of ZO-1 and occludin, along with enhanced BBB permeability was observed in cultured endothelial cells challenged with amyloid peptide Aβ1–42^58^. Similarly, occludin and ZO-1 expression was decreased following experimental induction of cerebral embolism in isolated rat brain capillaries^59^. While the exact mechanisms by which occludin and ZO-1 regulate the BBB are still being investigated, their roles in maintaining the selective permeability of the barrier are evident. Understanding the dysregulation of these proteins in C3GnT^−/−^ mice can provide valuable insights into the pathogenesis of various neurological disorders involving the gut-brain axis and potentially lead to the development of novel therapeutic strategies.

Taken together, our results suggest that an altered mucin glycosylation in the colon may contribute to a potential modulation of the phenotype of immature granule cells expressing PSA-NCAM. Interestingly, the long-term supplementation of the gut symbiont *Akkermansia muciniphila* in mouse model of early AD, resulted in a repair of the intestinal barrier dysfunction and ameliorations in cognition and anxiety-related behaviours, supporting a role for an impaired mucus barrier in the alteration occurring in the hippocampus of these mice^60^. Our behavioural studies confirmed an impaired hippocampal function in the C3GnT^−/−^ mice, as showed by the decline in recognition and spatial memory but more work is needed to pinpoint the microbial metabolites affecting the physiological and functional changes observed in the hippocampus of C3GnT^−/−^ mice.

## Materials and methods

### Study approvals

All experimental procedures and protocols performed were reviewed and approved by the University of East Anglia Animal Welfare and Ethical Review Body (UEA AWERB) and were conducted in accordance with the specification of the United Kingdom Animal Scientific Procedures Act, 1986 (Amendment Regulations 2012) under the Home Office project licence PP9417531. Reporting of the study outcomes comply with the ARRIVE (Animal Research: Reporting of In Vivo Experiments) guidelines^61^.

### Generation of C3GnT^−/−^ and C3GnT^+/+^ littermates

We first bred C57BL/6 mice with the C3GnT^−/−^ mice to generate mice heterozygous for the gene (C3GnT^+/−^). Once the pups (F1) reached sexual maturity (~ 6 to 8 weeks old), males and females C3GnT^+/−^ were bred to generate the F2 mouse colony. The F2 litters were composed of 25% homozygous wild-type (C3GnT^+/+^), 50% heterozygous C3GnT^+/−^ and 25% homozygous knock-out (C3GnT^−/−^) mice. After weaning (at ~ 3 weeks old), the mice litters were genotyped by analysis of genomic DNA (gDNA) from ear biopsies. Briefly, gDNA was extracted from ear biopsies using Quick Extract DNA extraction solution (Lucigen) following supplier’s advice. PCR was carried out using the GO TAQ (R) hot start polymerase (Promega, UK), with primers used at 1 µM final concentration (Table S2). The conditions consisted of an initial 2 min 95 °C denaturation step followed by 30 cycles of 94 °C for 45 s, 58 °C for 45 s, 72 °C for 45 s, and a final extension step of 5 min at 72 °C. The PCR products were analysed by electrophoresis on 1.5% agarose gels in 1× Tris–Acetate EDTA (TAE) for 60 min at 80 V in the presence of 100 bp DNA markers (New England Biolabs, USA). DNA was stained by adding 1 μL of Midori green direct DNA stain (Geneflow, UK) to 9 μL of PCR product prior to loading on the gel and imaged under UV light using an Alphaimager.

The C3GnT^+/+^ and C3GnT^−/−^ mice were then individually caged for 4 weeks (isolation period), in order to allow the development of the gut microbiota according to the genotype of mice coming from the same breeding pairs (littermate-control). Faecal samples were collected before the isolation time (~ 21 days after birth referred to as ‘Time 1’) and after the isolation period (~ 75 days after birth referred to as ‘Time 2’). When the mice reached ~ 9 to 10 weeks old, they were culled by raising concentration of CO_2_ and, after confirmation of death by dislocation of the neck, the brain, blood, caecal content, scraped mucus from colon were collected and stored for downstream analyses. C3GnT^+/+^ (n = 8) and C3GnT^−/−^ (n = 8) mice were also used for the battery of behavioural tests (Fig. S9).

### DNA extraction

DNA was extracted from faecal samples and scraped colonic mucus using the Fast DNA™ SPIN kit for Soil DNA extraction (MP Biomedicals, USA) with the following modifications. The weight of faecal material was measured in tared tubes. The samples were resuspended in 978 μL of sodium phosphate buffer (provided) before being incubated at + 4 °C for 1 h following addition of 122 μL of lysis solution MT Buffer. The samples were then transferred into the lysing tubes and homogenised in a FastPrep® Instrument (MP Biomedicals) 3 times for 40 s at a 6.0 m/s speed with 5 min interval on ice between each bead-beating step. The protocol was then followed as recommended by the supplier.

### 16S rDNA sequencing

Following extraction of DNA from faecal pellet and mucus, the concentration and quality of DNA was assessed by Qubit and Nanodrop. DNA was normalised to 5 ng/μL and sequenced in-house. The V3 and V4 16S rDNA gene regions were amplified using universal primers. For the first PCR, each well contained 4 μL kapa2G buffer, 0.4 μL dNTPs, 0.08 μL kapa2G polymerase, 0.4 μL 10 μM forward tailed specific primer, 0.4 μL 10 μM reverse tailed specific primer, 13.72 μL PCR grade water and 1 μL normalised DNA. The PCR conditions were 95 °C for 5 min followed by 30 cycles of 95 °C for 30 s, 55 °C for 30 s and 72 °C for 30 s with a final step at 72 °C for 5 min. A 0.7× SPRI using KAPA Pure Beads (Roche, UK) was then performed and the DNA was eluted in 20 μL of EB (10 mM Tris–HCl).

Following the first PCR, a second PCR was performed using 5 μL of the clean PCR product, 4 μL kapa2G buffer, 0.4 μL dNTPs, 0.08 μL kapa2G polymerase, 2 μL of each P7 and P5 primers of Nextera XT Index Kit v2 index primers (Illumina Catalogue No. FC-131-2001 to 2004) were added to each well. Finally, the 5 µL of the clean specific PCR mix was added and mixed. The PCR was run using 95 °C for 5 min, 10 cycles of 95 °C for 30 s, 55 °C for 30 s and 72 °C for 30 s followed by a final step at 72 °C for 5 min. The obtained libraries were then quantified using the Quant-iT dsDNA Assay Kit, high sensitivity kit (Fisher Scientific, UK) on a FLUOstar Optima plate reader. Libraries were pooled following quantification in equal quantities. The final pool was cleaned with 0.7× SPRI using KAPA Pure Beads. The final pool was quantified on a Qubit 3.0 instrument and run on a High Sensitivity D1000 ScreenTape (Agilent) using the Agilent Tapestation 4200 to calculate the final library pool molarity.

The pool was run at a final concentration of 8 pM on an Illumina MiSeq instrument using MiSeq® Reagent Kit v3 (600 cycles, Illumina) following the Illumina denaturation and loading recommendations which included a 20% PhiX spike in (PhiX Control v3 Illumina). The raw data were analysed locally on the MiSeq using MiSeq reporter.

Raw reads produced by Illumina MiSeq sequencing of the 16S amplicons have been processed with the Dadaist2 0.8 workflow CIT^62^, using SeqFu 1.9 CIT^63^ to remove the universal primers used to amplify the target region, and then processed via DADA2 1.16^64^ to identify the Amplicon Sequence Variants (ASVs) and to generate a contingency table (with the raw abundance of each ASV in each sample). The DECIPHER package^65^ has been used to assign taxonomy to each ASV identified using the SILVA 138 database^66^, and the results have been exported to a PhyloSeq CIT^67^ object for downstream analysis.

### RNA extraction and RT-qPCR

Mouse colonic tissue and brain were harvested and stored in RNAlater at + 4 °C until RNA extraction. Samples (10–100 mg) were then transferred in a tube containing 1 mL of QIAzol Lysis Reagent (Qiagen, UK) and stainless-steel beads of 5 mm (Qiagen, UK). Homogenisation was achieved using the FastPrep®-24 by 2 intermittent runs of 60 s at 4.0 m/s speed with 5 min interval at room temperature. RNA extraction was performed using the RNeasy Lipid Tissue Mini Kit (Qiagen) following the manufacturer’s instructions for purification of total RNA from animal tissues. Elution was performed as recommended with 50 μL RNAse-free water. The quality and concentration of the RNA samples was assessed using the NanoDrop 2000 spectrophotometer, the Qubit RNA HS assay on Qubit® 2.0 fluorometer (Life Technologies, UK) or Agilent RNA 600 Nano kit on Agilent 2100 Bioanalyzer (Agilent Technologies, Stockport, UK). Following RNA extraction, cDNA synthesis was carried out using QuantiTect Reverse Transcriptase (Qiagen) according to the manufacturer’s instructions including controls lacking the transcriptase to test for DNA contamination (-RT control).

For quantitative reverse transcription PCR (RT-qPCR) reactions were performed using SYBR green detection technology on the Roche light cycler 480 (Roche Life Science, UK). The samples were loaded into 384 microtiter plates in a randomised way and the primers were also randomised across triplicates. Primer sequences are given in Table S3.

### Soluble mucin extraction and purification

The mucus was scraped from the colon and resuspended in cold PBS and kept in ice. The solution was then centrifuged at 1000×*g* for 30 min to separate bacteria (pellet) and soluble proteins (supernatant). The bacteria pellet was stored at − 80 °C until DNA extraction. The presence of Muc2 in the soluble fraction was confirmed by slot blot, using an anti-MUC2 antibody, after blocking the PVDF membrane with Pierce™ Protein-Free (PBS) Blocking Buffer (ThermoFisher). The supernatant from samples from 15 C3GnT^+/+^ and 12 C3GnT^−/−^ were pooled together according to the genotype and processed for mucin extraction and purification. Briefly, caesium chloride was added to the samples at a final density of 1.4 g/mL and the samples were ultracentrifuged at 42,000 rpm on a Beckman Coulter 70 Ti rotor. Fractions (1 ml each) were collected and those with a density higher than 1.4 g/mL were pooled together, dialysed against water and freeze-dried. The purified mucins were subjected to β-elimination under reductive conditions (0.1 M NaOH, 1 M sodium borohydride, NaBH_4_) for 20 h at 45 °C. The reaction was stopped by slowly adding 5 drops of glacial acetic acid (Sigma). A desalting column was assembled by packing a Paster pipette, with Glass Wool to control the flow (Sigma) and Dowex 50 W × 8 hydrogen form beads (Sigma). Following elution of the desalting column with 15 mL of glacial acetic acid, the samples, were loaded onto the column. The collected fractions were dried under a stream of nitrogen to evaporation the excess of borates.

Permethylation was performed on released *O*-glycans from mucin samples. Samples were solubilised in 200 μL dimethyl sulfoxide (DMSO). Then NaOH (1 pellet) and 300 μL iodomethane were added to the suspension in anhydrous conditions and the samples vigorously shaken at room temperature for 90 min. The permethylation reaction was stopped by addition of 1 mL acetic acid (5% vol/vol). Permethylated *O*-glycans were extracted in 1–2 mL of chloroform and 5 ml of ultrapure water, mixed thoroughly by vortexing and centrifuged at 14,000×*g* for 2 min to allow the mixture to set into two layers. The upper aqueous part (containing DMSO) was removed and discarded. The lower chloroform layer was washed five times with MilliQ water (1 mL) and then dried under nitrogen using an evaporator unit. The dried samples were dissolved in 30% acetonitrile in 0.1% aqueous trifluoroacetic acid (TFA, Sigma) and mixed 1:1 with 20 mg/mL 2,5-dihydroxy-benzoic acid (DHBA) in the same solvent. 2 μL were spotted on a matrix assisted laser desorption ionization (MALDI) target plate for analysis.

### MALDI-ToF MS mucin glycosylation analysis

MALDI-TOF (Tandem time of flight) and TOF/TOF–MS data were acquired using the Bruker Autoflex analyzer mass spectrometer (Applied Biosystems, Foster City, CA, US) in the positive-ion and reflectron mode. The relative quantification of sialylation on mucins was calculated based on the sum of all areas of mass peaks corresponding to sialylated structures divided by the sum of all areas of mass peaks corresponding to defined* O*-glycans. Similar calculation was done to determine the relative quantification of fucosylation or sulfation on mucins. The identification of glycan structures was conducted using *Glycoworkbench* software^68^.

### FITC-dextran in vivo permeability assay

To assess intestinal permeability, C3GnT^+/+^ and C3GnT^−/−^ mice were fasted for 4 h before administration of 150 μL of fluorescein isothiocyanate dextran (FITC) (80 mg/mL 4 kD; Sigma-Aldrich) in sterile 1× PBS by oral gavage. Mice were culled following schedule-1 by raising concentration of CO_2_ 4 h following the FITC treatment, and blood was collected by intracardial puncture. The blood samples were diluted 1:4 in PBS and the concentration of the FITC-dextran was determined using a fluorimeter (FLUOstar OPTIMA, BMG LABTECH) with an excitation wavelength at 490 nm and an emission wavelength of 520 nm. Serial-diluted FITC-dextran was used to generate a standard curve (from 8000 to 0.125 ng/mL) (Fig. S3).

### Metabolomics

The caecal content (100–200 mg) from C3GnT^+/+^ (n = 6) and C3GnT^−/−^ (n = 6) mice, the brain (100–200 mg) from C3GnT^+/+^ (n = 9) and C3GnT^−/−^ (n = 9) mice and the serum (50–60 µL) pooled from C3GnT^+/+^ (n = 5) and C3GnT^−/−^ (n = 5) mice were processed and analysed by Metabolon, Inc, USA. A total of 705 metabolites were detected in the brain, 911 in the caecal contents and 945 detected the serum. Metabolites were identified by comparison to library entries of purified standards or recurrent unknown entities. Metabolon maintains a library based on authenticated standards that contains the retention time/index (RI), mass to charge ratio (*m/z)*, and chromatographic data (including MS/MS spectral data) on all molecules present in the library. Furthermore, biochemical identifications are based on three criteria: retention index within a narrow RI window of the proposed identification, accurate mass match to the library ± 10 ppm, and the MS/MS forward and reverse scores between the experimental data and authentic standards. The MS/MS scores are based on a comparison of the ions present in the experimental spectrum to the ions present in the library spectrum.

### Free floating immunolabelling of brain sections

Following transcardial flushing with 4% paraformaldehyde (PFA) in PBS, mouse brains were extracted and kept overnight in PFA at + 4 °C. Next, the brains were washed in PBS and dehydrated by incubation in an ascending alcohol series, starting from 30–50–70–90 to 100% of ethanol for 1 h each, followed by hydration in decreasing concentrations of ethanol, from 100 to the 30% and using PBS in the final step. The brains were then embedded in 3% agar with the bulbs facing the top and 60 µm slices were cut using a vibratome (Leica, VT1200S). The floating brain sections were then transferred into a multi-well plate (Corning, UK) with a paintbrush. For immunohistochemistry, free floating sections were selected according to the region of interest, following a mouse brain atlas (Paxinos and Franklin's the Mouse Brain in Stereotaxic Coordinates, Compact 5th Edition).

Brain sections (Bregma coordinates from − 1.22 to − 2.46 mm, including hippocampus and hypothalamus) were first incubated with antigen unmasking buffer (10 mM citrate buffer, 0.05% Tween 20, pH 6.0) pre-warmed at 70 °C for 15 min at 70 °C in a water-bath. The sections were then blocked for 2 h with a PBS solution containing 20% normal goat serum (NGS, Gibco) and 1% Triton X100 and then incubated overnight at + 4 °C with primary antibodies (Table S4) in a solution containing 0.2% NGS and 0.1% Triton X100. The sections were then washed five times by incubation of 1 h each at room temperature in 0.2% NGS and 0.1% Triton X100 and then incubated overnight at + 4 °C with the relevant secondary antibodies diluted in the buffer used for the primary antibodies (Table S4). Following washing in PBS (6 times, 30 min incubation each), the sections were stained with 4ʹ,6-diamidin-2-fenilindolo DAPI (1 µg/mL, Thermo Fisher, UK) for 5 min, washed, mounted and cover-slipped with mounting medium (VECTASHIELD Antifade Mounting Medium-H100).

### Western blot analysis

Fresh extracted brains from C3GnT^+/+^ and C3GnT^−/−^ littermates were separated in the two hemispheres to get sagittal sections and stored at − 80 °C until use; for the tissue lysis then the exposed hippocampal area was excised with a blade from the right and left hemisphere, resulting in the hippocampus and surrounding regions. The tissue extracts (15–20 mg) were homogenized in 500 µL of lysis buffer (20 mM Tris–HCl, pH 8.0, 150 mM NaCl, 5 mM EDTA, 1 mM phenylmethylsulphonyl fluoride, 70 µg/mL aprotinin, 10 µg/mL leupeptin, 2% Triton X-100) as described^69^. Lysates were treated with 25 ng/µL endosialidase for 45 min on ice. The proteins were then separated by 4–12% SDS-PAGE (50 µg of total protein per lane), followed by Western blotting for 16 h at 4 °C. Membranes were incubated with 0.4 µg/mL of NCAM-specific rat monoclonal antibody (mAb) H28 and 1 µg/mL of polySia-specific mouse mAb 735 (IgG2a) (kindly provided by Prof Herbert Hildebrandt). To identify tight junction proteins, ZO-1-specific rabbit polyclonal antibody (PABs) 1:1000 (Thermo Fischer Scientific) and Occludin-specific mouse mAb 1:1000 (Thermo Fischer Scientific) were used. GAPDH was used as loading control using a GAPDH- specific PABs (Abcam). Bound antibodies were detected with peroxidase-coupled anti-mouse or anti-rabbit IgG (Vector Laboratories) and developed by enhanced chemiluminescence using Clarity Western ECL Substrate (BioRad). Quantification was carried out using ImageLab software (Bio-Rad).

### Behavioural analysis

The novel object recognition (NOR), a measure of recognition memory, was performed as described previously^70,71^ with slight modifications. Briefly, on day 1, mice (n = 8/group) were habituated in a grey 50 × 50 × 50 cm apparatus illuminated with low lux (100 lx) lighting, by being placed into the empty maze and allowed to move freely for 10 min then returned to the cage. On day 2, mice were placed back in the maze and conditioned to a single object for a 10 min period. On day 3, mice were placed into the same experimental area in the presence of two identical objects for 15 min, after which they were returned to their respective cages and an inter-trial interval of 1 h was observed. One of the familiar objects was replaced with a novel object. Mice were placed back within the testing area for a final 10 min. Videos were analysed for a 5 min period, after which, if an accumulative total of 8 s in the presence of both objects (familiar and novel) failed to be reached, the analysis continued for the full 10 min or until the mouse would complete 8 s of time spent close to the object. The mice not reaching the required time were excluded from the analysis^72^. A discrimination index (DI) was calculated as follows: DI = (TN − TF)/(TN + TF), where TN is the time spent exploring the novel object and TF is the time spent exploring the familiar object.

The Y-maze spontaneous alternation test, a measure of spatial working memory, was performed on the final day of behavioural testing, as previously described^73^. Briefly, the Y-maze apparatus made of white Plexiglas of three distinct arms (dimensions 38.5 × 8 × 13 cm, each arm at a 120° angle from each other) was illuminated with low lux (100 lx) lighting. The mouse to test was placed in the maze and allowed to explore freely for 7 min whilst tracking software recorded zone transitioning and locomotor activity (Ethovision XT, Noldus, UK). It is expected that the mouse following the natural exploratory instinct, goes around the maze and visits each arm equally.

The open field test (OFT) was conducted as previously described^74^. Briefly, the mouse to be tested was placed in the centre of the OFT and a video tracking system (Ethovision XT, Noldus, UK) recorded the total distance the mice travelled, as well as with the time they spent in the centre of the field within the first 5 min. The open field maze was cleaned between each mouse with 20% ethanol to eliminate odours. It is expected that the mouse goes around exploring the box, staying close to walls (thigmotaxis)^42^.

### Statistics

All statistical analyses were conducted using the R version 4.1.0. In the glycan analysis from intestinal mucins, at least three technical replicates were performed for each fraction and t-tests were used to compare carried out for each fraction between C3GnT^+/+^ and C3GnT^−/−^ littermates.

In the analysis of the gut microbiota, 16S data were filtered such that only ASVs with count greater than 3 in at least 20% of samples were included. ASVs were then aggregated into families for primary analysis. The relative abundances and centred-log ratios of each family were plotted by genotype with means and confidence intervals.

Bray–Curtis distance matrix was calculated based on total sum scaled data, and multidimensional scaling (MDS) ordination was plotted in strata corresponding to sample point (Time 1 vs Time 2). PERMANOVA was conducted using adonis2 from the vegan R package. For differential abundance analysis, two approaches were used. First, DESeq2 was applied on the count data (aggregated at family level) separately for faecal samples (Time 2) and scraped mucus independently to compare family counts across genotypes. All DESeq2 defaults were accepted, including Benjamini–Hochberg correction to p-values.

Second, standard linear regression models were applied to centred-log ratios calculated using the microbiome package in R. Analyses were also repeated on three other datasets: (1) all ASVs, (2) top 20 ASVs only aggregated into families, (3) data aggregated to Phylum level.

Differences in gene expression between groups were calculated by estimating the effect of genotype on Ct values, adjusting for the Ct values of both housekeeping genes (*Gapdh* and *Tbp*), with individual mouse included as a random effect and replicate as a fixed effect. The data from three samples were removed prior to analysis, as outliers based on visual inspection of the Ct values. Data are presented as estimated ratio of gene expression (C3GnT^−/−^ vs C3GnT^+/+^) with 95% confidence intervals for each gene. P-values were adjusted using the Benjamini–Hochberg method taking into account fourteen genes being simultaneously tested.

An estimate of the false discovery rate (*q*-value) was calculated to take into account the multiple comparisons that normally occur in metabolomic-based studies. The *q*-value describes the false discovery rate; a low *q*-value (*q* < 0.10) is an indication of high confidence in a result.

In the behavioural study, data were analysed using the “Origin 9” software (OriginLab, Northampton, USA). Unpaired t-test with normality checks of QQ-plots were used for the assessment of behavioural tests in mice. Outliers were detected by ROUT or Grubbs test.

### Supplementary Information

Supplementary Information.

## Data Availability

The authors confirm that the data supporting the findings of this study are available within the article and its Supplementary Materials. The raw reads produced for the metabarcoding analysis of this project are available at the EBI ENA, under Project Accession ID PRJEB57587 and study ID ERP142578. The metabolomics data will be made available on demand to the corresponding author.
