# Supplementary material for: Role of mucin glycosylation in the gut microbiota-brain axis of core 3 O-glycan deficient mice
Source: Sci Rep. 2023 Aug 26;13:13982. doi: 10.1038/s41598-023-40497-8 (PMC10460388; doi:10.1038/s41598-023-40497-8)
Supplement: Supplementary file 1 — Supplementary Information. [file 41598_2023_40497_MOESM1_ESM.docx]

**Supplementary figures:**

**A B**


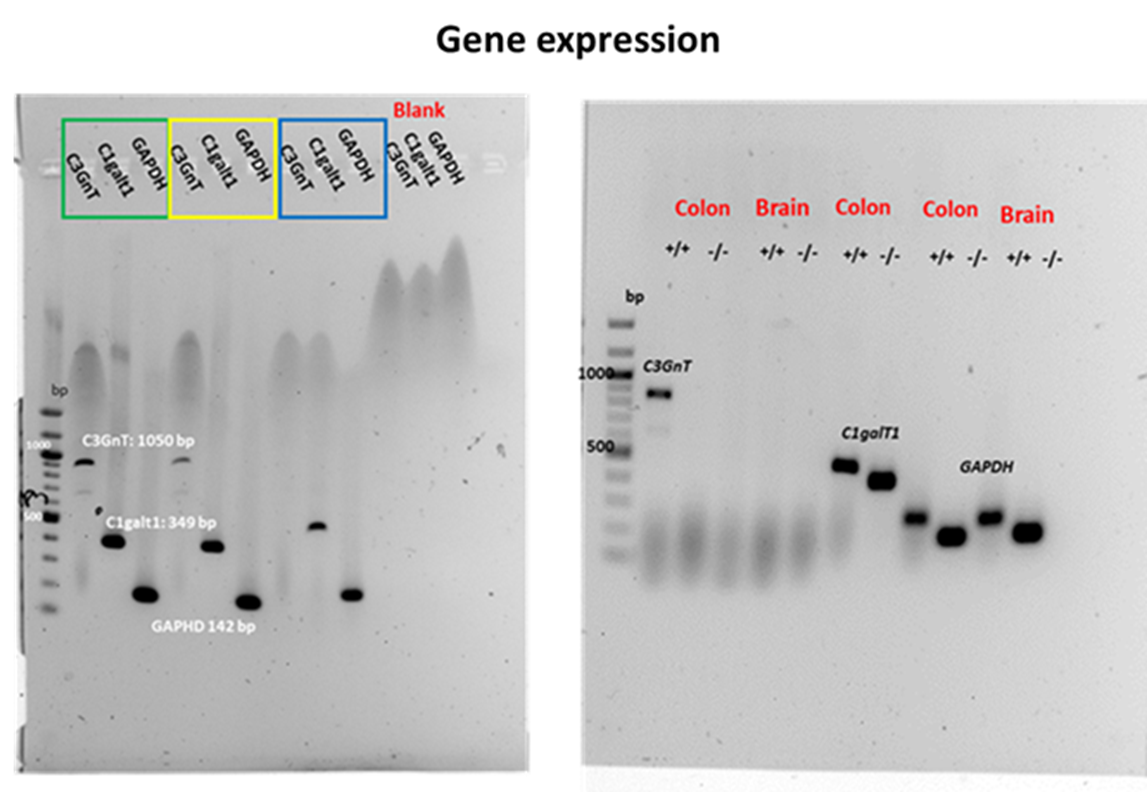


**Figure S1. RT-PCR from colon and brain of C3GnT^+/+^ and C3GnT^-/-^ mice.** (**A**) The agarose gel confirmed that *C3GnT* gene is expressed mainly in the proximal colon (green box) of mice and its expression decreases towards the middle (yellow box) and distal (blue box) part of the colon. (**B**) *C3GnT* gene (~ 1050 bp) is not expressed in the brain of C3GnT^+/+^ and C3GnT^-/-^ mice. C1galT1 (~ 349 bp) and GAPHD (~ 142 bp) were used as positive controls in the brain and colon cDNA samples of C3GnT^+/+^ and C3GnT^-/-^ mice.


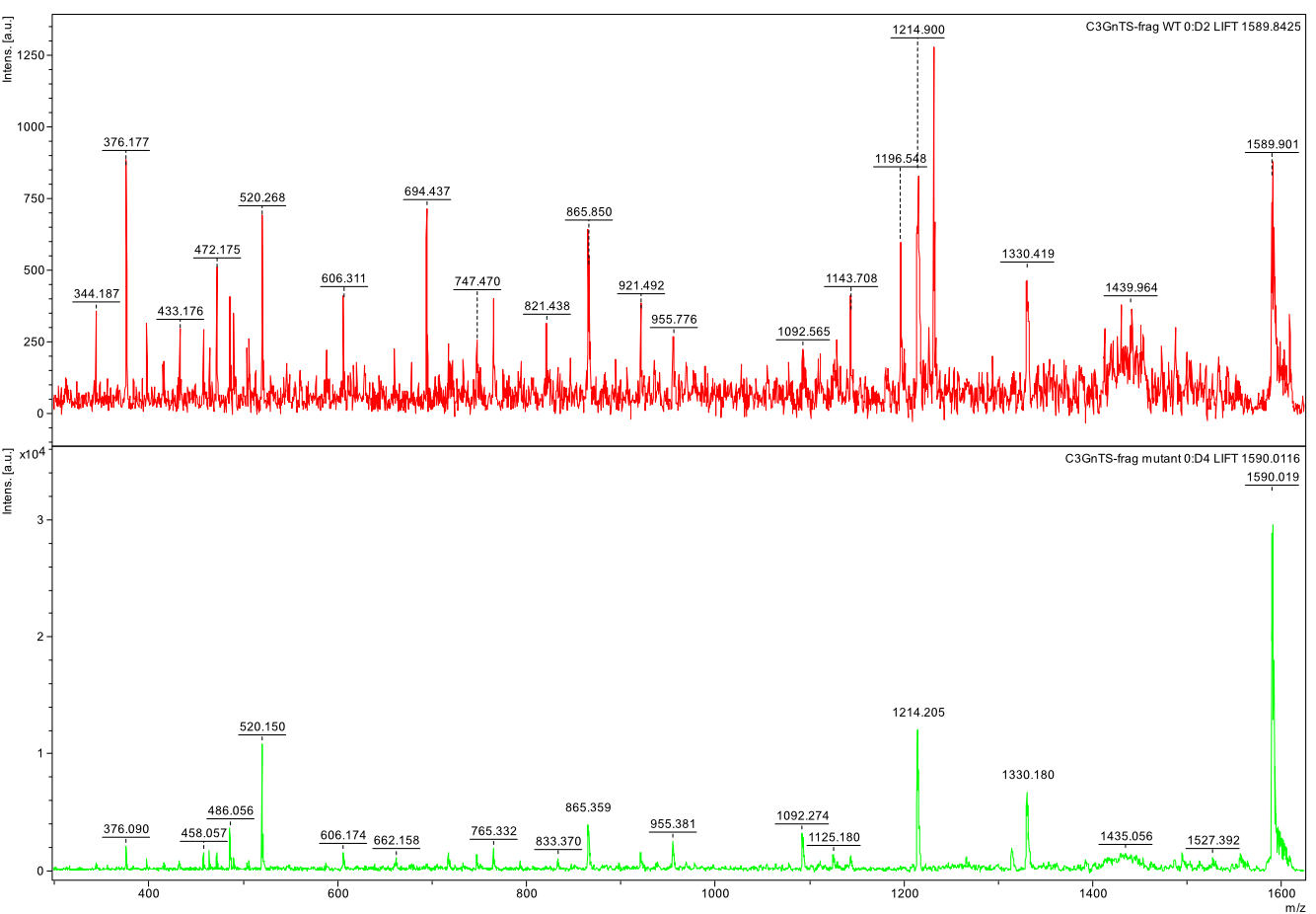


**Figure S2. Fragmentation spectra of the m/z peak at 1589, corresponding to Neu5Ac1Hex2HexNAcGalNAcol from C3GnT^+/+^ and C3GnT^-/-^ littermates.** The spectra obtained for C3GnT^+/+^ (red) and C3GnT^-/-^ (green) indicate differences in the structural composition of the mucin glycans.


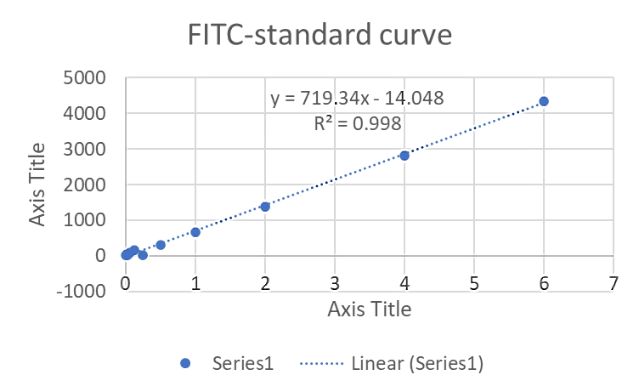


**

**

**Figure S3. FITC-dextran assay in C3GnT^-/-^ and C3GnT^+/+^ littermate.** The FITC-dextran standard curve (from 5 µg/mL to 0.125 µg/mL) (left panel) was used to calculate the concentration of FITC-dextran concentration (µg/ml) measured in the plasma of C3GnT^-/-^ (n = 3) and C3GnT^+/+^ (n = 3) mice 4 h after oral gavage (right panel).


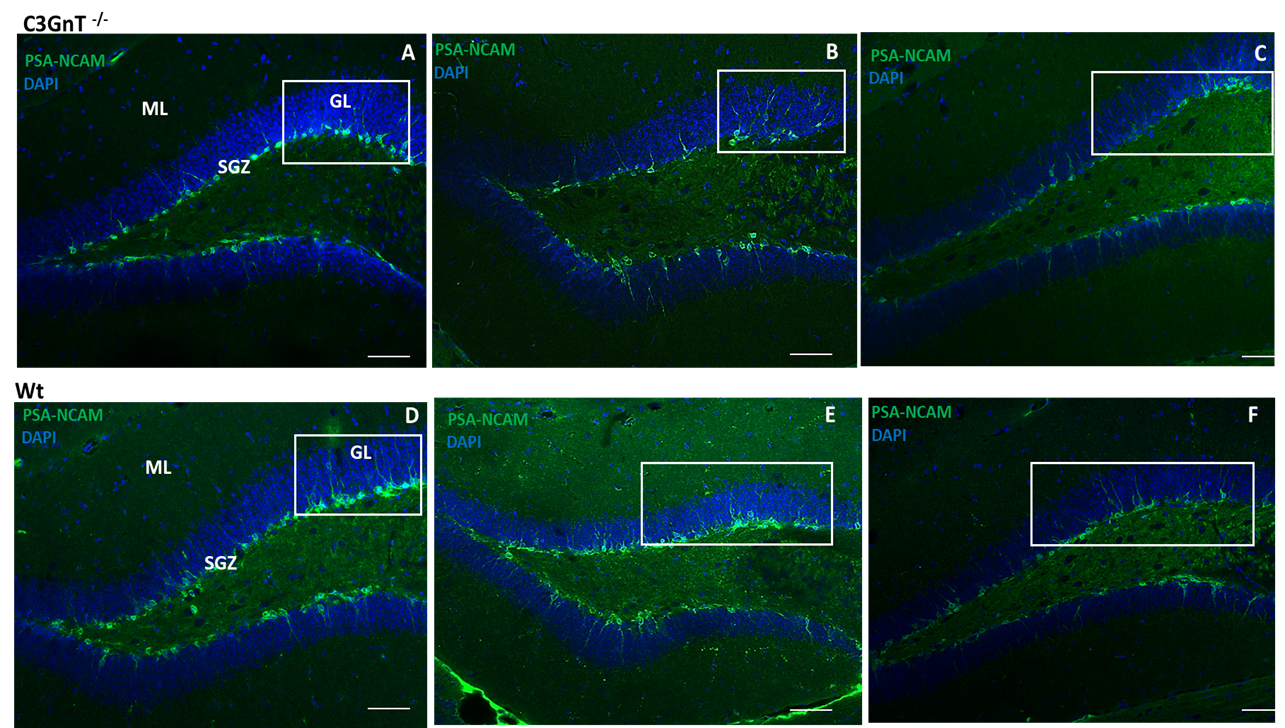


Figure S4. Dorso-ventral PSA-NCAM expression in the DG of C3GnT^-/-^ and WT mice. Immunohistochemistry images showing PSA-NCAM^+^ cells (green) in the DG of C3GnT^-/-^ (n = 4) and WT mice (n = 4). The PSA-NCAM^+^ granule cells are located in the subgranular zone (SGZ), the innermost area of the granule cell layer (GL) (white box) where the dendrites are projecting towards the upper molecular layer (ML). Cell nuclei are counterstained with DAPI (blue). (A-B-C) Immunohistochemistry images showing the characteristic PSA-NCAM aberrant expression of granule cells in the C3GnT^-/-^ mice from dorsal toward the ventral DG. (D-E-F) Immunohistochemistry images showing the characteristic PSA-NCAM expression of granule cells from dorsal toward the ventral DG of WT mice. Bregma position: -1.46 mm (A-D), -1.58 mm (B-E), -2.06 mm (C-F). Images: objective 10X, scale bar: 100 µm.


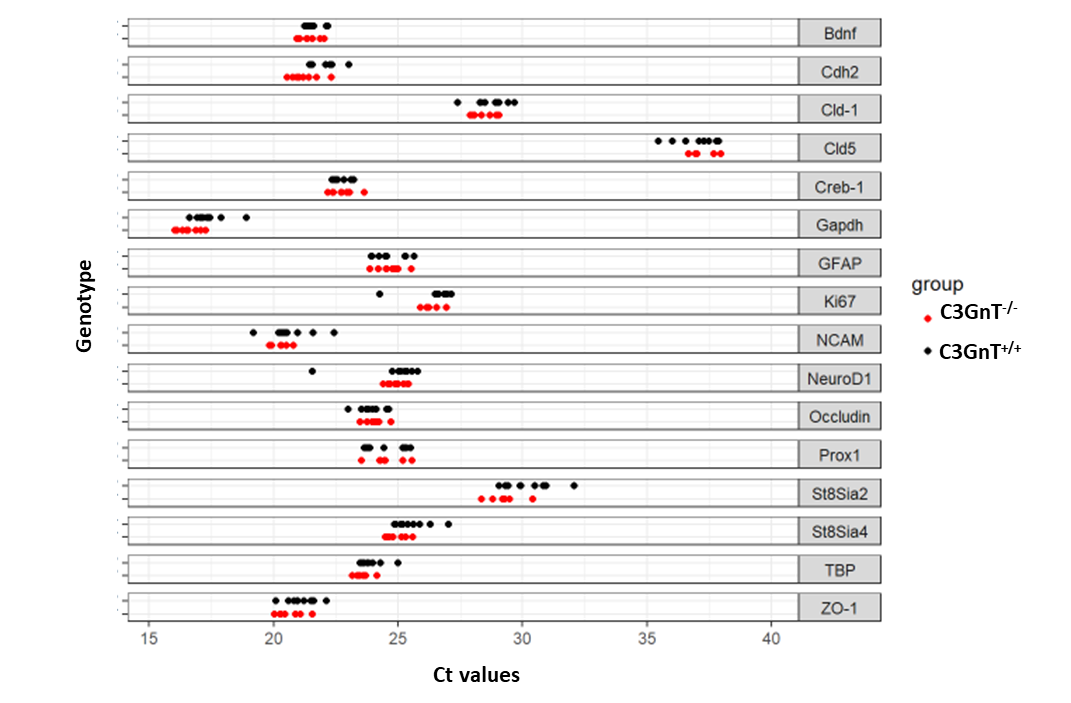


**Figure S5. Gene expression from brain of C3GnT^+/+^ and C3GnT^-/-^ mice.** Points represent mean Ct values for each individual animal. No correction for reference genes is made, but Ct values for reference genes *Tbp* and *Gapdh* are shown.


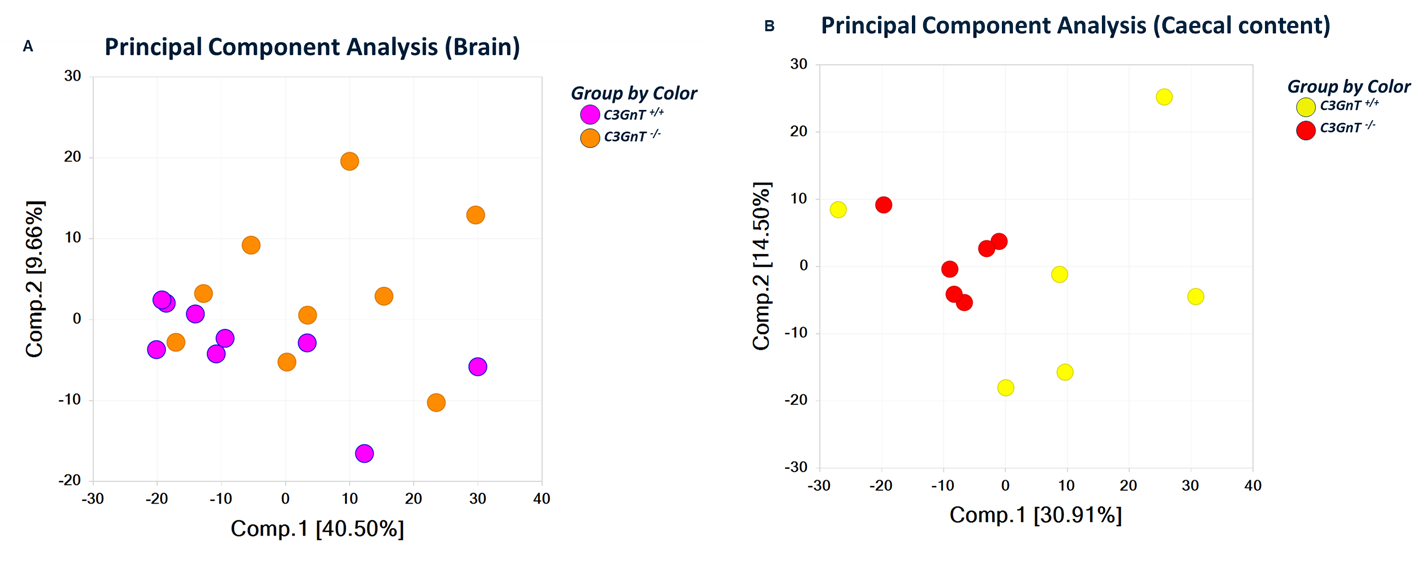


**Figure S6. Metabolic profile from brain and caecal content of C3GnT^+/+^ and C3GnT^-/-^ littermates. (A**) The principal component analysis (PCA) results for the brain showed some separation of the C3GnT^+/+^ and C3GnT^-/-^ samples, with the C3GnT^+/+^ samples mostly separating toward the middle/bottom of component 2, while the C3GnT^-/-^ samples separated mostly toward the top. (**B**) The PCA plot for the caecal content revealed more distinctive segregation of C3GnT^-/-^ samples away from C3GnT^+/+^ samples, with most of the C3GnT^-/-^ samples segregating to the left on component 1.


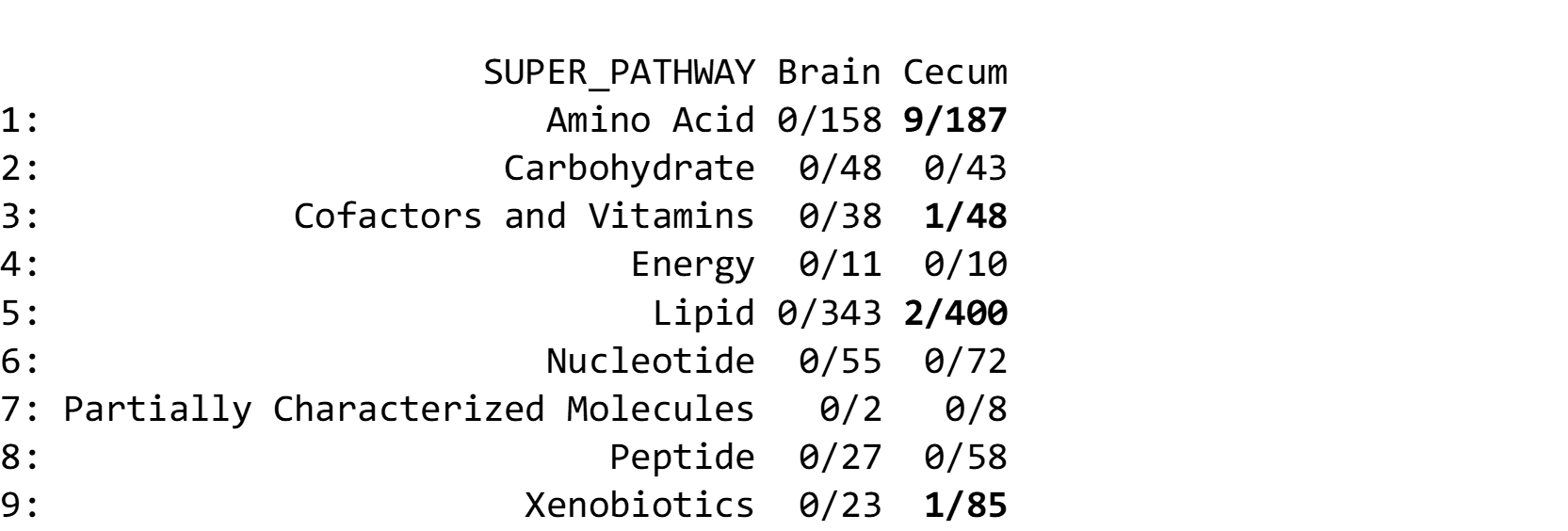


**Figure S7. Statistically significant changes in metabolites in the caecum of C3GnT^+/+^ and C3GnT^-/-^ mice.** Number of metabolites with statistically significant differences (at q<0.05 after Benjamini-Hochberg correction) between C3GnT^+/+^ and C3GnT^-/-^ mice in the brain and cecum organised by super pathway. There were no significant differences between metabolites in the brain, nine of the 13 ‘significant’ metabolites in the caecum belong to the amino acid super pathway.


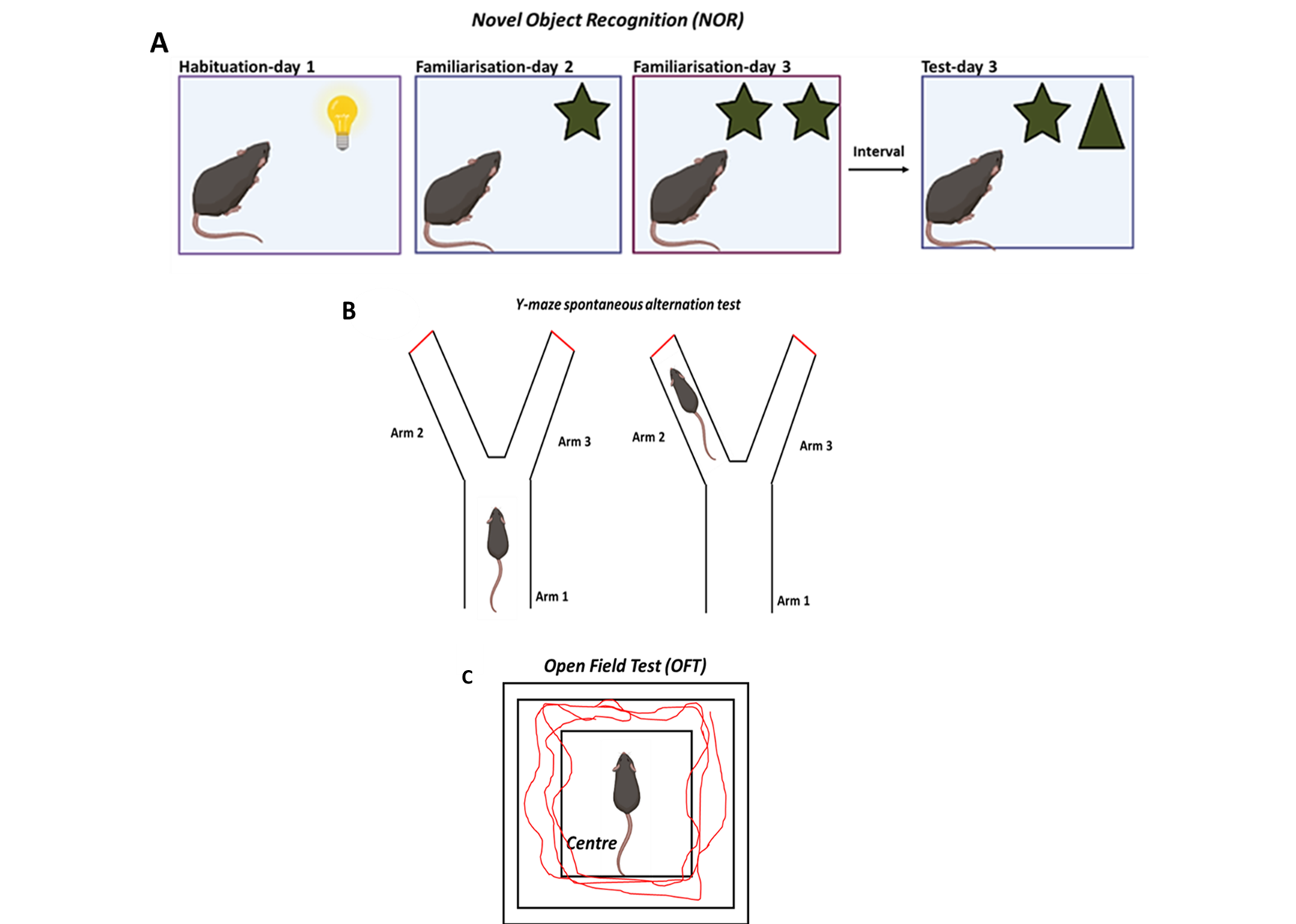


**Figure S8. Schematic illustrations showing the behavioural tasks administered to the C3GnT^+/+^ and C3GnT^-/-^ mice.** (**A**) On day 1, mice (one at the time) were habituated into the maze, on day 2, the mice were exposed to a single object and on the day 3, they were conditioned to two identical objects before one of the objects was replaced by a novel one for the test. (**B**) After introduction to the centre of the maze, the animals (one at the time) were allowed to freely explore the three arms. (**C**) The mice (one at the time) were placed in the centre of the box and free to move from it.


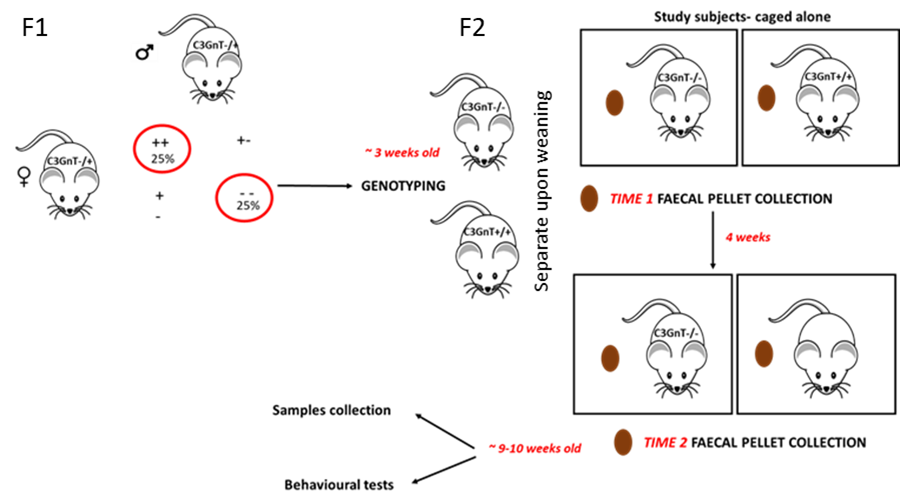


**Figure S9.** **Breeding scheme used for the generation of C3GnT^+/+^ and C3GnT^-/-^ littermates to evaluate the impact of host genetic on the gut microbiota composition.** Heterozygous C3GnT^-/+^ mice were crossed together in the F1 generation, generating the F2 litters covering 25% wild type (C3GnT^+/+^), 50 % heterozygous (C3GnT^+/-^) and 25% homozygous C3GnT^-/-^ mice in the same cage. At around 3 weeks old, the mice were weaned and genotyped, then separated according to their genotype and caged alone for 4 weeks. Faecal pellets were collected at Time 1 (~ 21 days after birth) and Time 2 (~ 75 days after birth). When they reached ~ 9-10 weeks old, 27 mice (15 C3GnT^-/-^ and 12 C3GnT^+/+^) were culled and samples collected and 16 mice (8 C3GnT^-/-^ and 8 C3GnT^+/+^) underwent behavioural tests.

**Supplementary Tables:**

**Table S1. Log2 fold change of bacterial family abundance estimated using DESeq2 in faecal samples scraped mucus at 72 days.** Negative fold changes suggest higher abundances in the C3GnT^-/-^ mice compared to the C3GnT^+/+^ littermates. The top ten statistically significant families are shown for each sample point. Adjusted p-values are calculated using the Banjamini-Hochberg correction.

| **Sample point** | **Family** | **Base Mean** | **log2 Fold Change (WT vs KO)** | **p-value** | **Adjusted p-value** |
| --- | --- | --- | --- | --- | --- |
| faeces | *Lactobacillaceae* | 5350.81 | -1.66 | 0.0037 | 0.0846 |
|  | *Atopobiaceae* | 211.18 | -1.99 | 0.0745 | 0.8567 |
|  | *Sutterellaceae* | 553.12 | -1.01 | 0.1334 | 0.8893 |
|  | *Muribaculaceae* | 245.56 | -0.61 | 0.1694 | 0.8893 |
|  | *Eggerthellaceae* | 400.34 | -1.11 | 0.2423 | 0.8893 |
|  | *Anaerovoracaceae* | 30.29 | 1.43 | 0.2446 | 0.8893 |
|  | *Deferribacteraceae* | 920.97 | -0.96 | 0.2741 | 0.8893 |
|  | *unclassified_Bacteroidales* | 944.60 | -0.36 | 0.3608 | 0.8893 |
|  | *[Eubacterium] coprostanoligenes group* | 13.37 | 2.29 | 0.4342 | 0.8893 |
|  | *Erysipelatoclostridiaceae* | 13.09 | 1.20 | 0.4485 | 0.8893 |
| mucus | *Prevotellaceae* | 563.91 | -1.11 | 0.0213 | 0.3101 |
|  | *Lactobacillaceae* | 11009.26 | -1.57 | 0.0270 | 0.3101 |
|  | *Atopobiaceae* | 120.52 | -2.47 | 0.1225 | 0.8261 |
|  | *Sutterellaceae* | 691.22 | -1.03 | 0.1925 | 0.8261 |
|  | *Deferribacteraceae* | 1533.29 | -1.07 | 0.2173 | 0.8261 |
|  | *Clostridiaceae* | 54.24 | -1.20 | 0.2378 | 0.8261 |
|  | *Oscillospiraceae* | 1917.21 | 0.80 | 0.2971 | 0.8261 |
|  | *Rikenellaceae* | 2430.23 | 0.31 | 0.2993 | 0.8261 |
|  | *Tannerellaceae* | 6.60 | -1.56 | 0.3359 | 0.8261 |
|  | *Anaerovoracaceae* | 13.71 | 1.38 | 0.4547 | 0.8261 |

**Table S2. Primers used for PCR analysis of C3GnT genotype**

| **Primer name** | **Sequence** | **Target** |
| --- | --- | --- |
| C3GnT WT/KO_F | AGCCTGAGCCACCCTATCCAGTT | gDNA/Genotyping |
| C3GnT WT_R | AGCCAGTGTCGTGGGACTCTTG | gDNA/Genotyping |
| C3GnT KO_R | GACGACAGTATCGGCCTCAGGAAGA | gDNA/Genotyping |

**Table S3**: **List of primers used for qPCR in brain samples of C3GnT^-/-^ and C3GnT^+/+^ mice.**


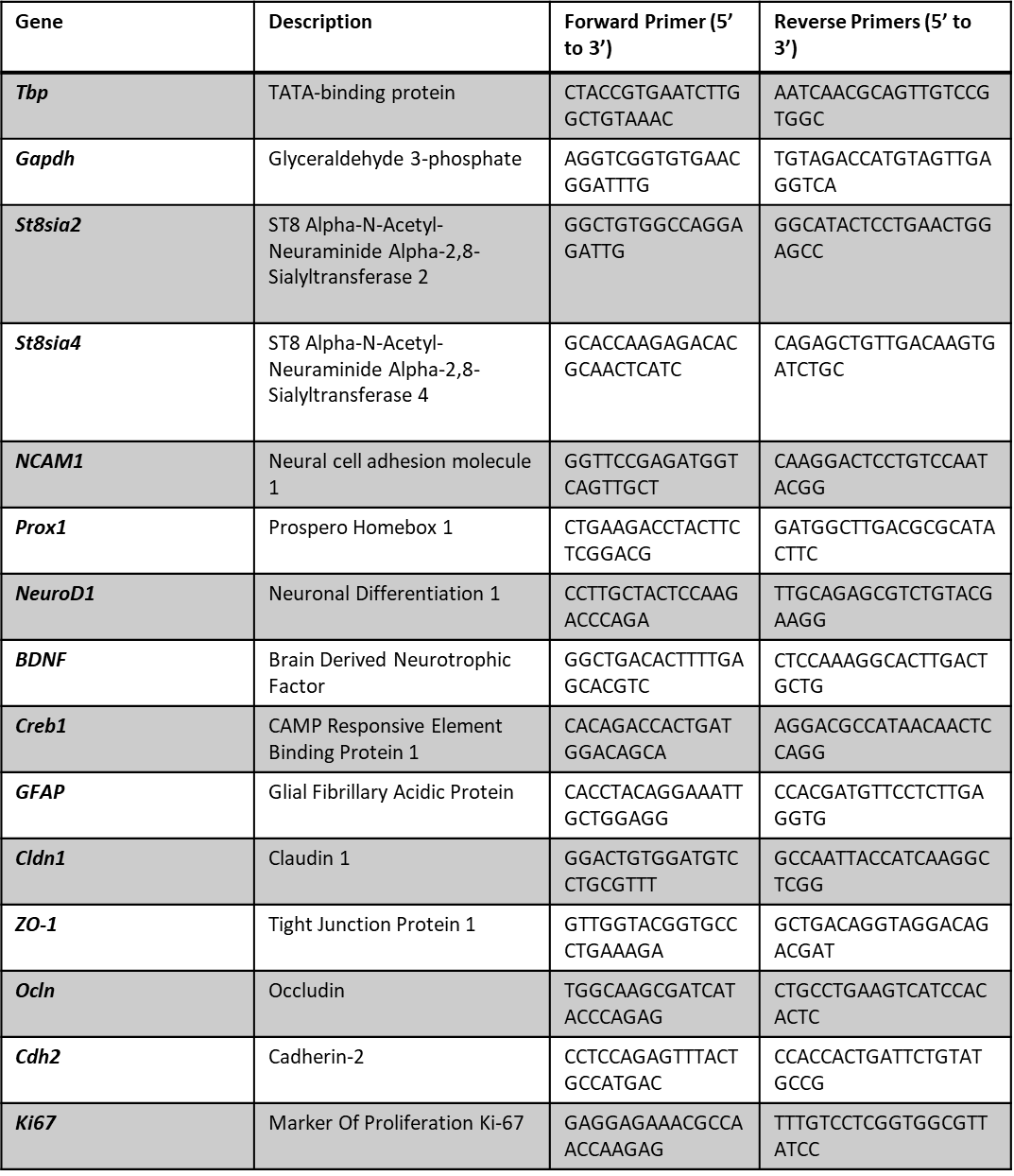


**Table S4**: **List of antibodies and dilution used in this study**

| **Primary antibody** | **Origin** | **Supplier** | **Dilution** |
| --- | --- | --- | --- |
| Polysialic Acid-NCAM (clone 2-2b) | Mouse | MAB5324 Merck | 1:200 |
| NCAM-specific monoclonal antibody (mAb) H28 | Rat | kindly provided by Prof Herbert Hildebrandt | 0.4 µg/mL |
| polySia-specific mAb 735 (IgG2a) | Mouse | kindly provided by Prof Herbert Hildebrandt | 1 µg/mL |
| ZO-1-specific polyclonal antibody (PABs) | Rabbit | 61-7300 Thermo Fisher Scientific | 1:1000 |
| Occludin-specific mAb | Mouse | 33-1500 Thermo Fisher Scientific | 1:1000 |
| GAPDH-specific PABs | Rabbit | ab9485 Abcam | 1:2000 |
| **Secondary antibody** | **Fluorophore**  **/ Enzyme** | **Supplier** | **Dilution** |
| Goat anti-Mouse IgG (H+L) Cross-Adsorbed | Biotin | 62-6540 Thermo Fisher Scientific | 1:250 |
| Streptavidin-conjugate | 488 | S11223 Thermo Fisher Scientific | 1:500 |
| Anti-mouse IgG | Peroxidase | Vector Laboratories |  |
| Anti-rabbit IgG | Peroxidase | Vector Laboratories |  |
